# Sleep Disorders and Stroke: Pathophysiological Links, Clinical Implications, and Management Strategies

**DOI:** 10.3390/medsci13030113

**Published:** 2025-08-05

**Authors:** Jamir Pitton Rissardo, Ibrahim Khalil, Mohamad Taha, Justin Chen, Reem Sayad, Ana Letícia Fornari Caprara

**Affiliations:** 1 Neurology Department, Cooper University Hospital, Camden, NJ 08103, USA; fornari-caprara-ana@cooperhealth.edu; 2 Faculty of Medicine, Alexandria University, Alexandria 5372066, Egypt; ietfeiheh2016@gmail.com; 3 School of Medicine, Texas A&M University Health Science Center, Bryan, TX 77807, USA; mohamadrtaha@gmail.com (M.T.); justinguy941@gmail.com (J.C.); 4 Faculty of Medicine, Assiut University, Assiut 71515, Egypt; reemoo.8527@gmail.com

**Keywords:** sleep disorder, insomnia, obstructive sleep apnea, sleep-disorder breathing, cardiovascular risk, cerebrovascular risk, stroke

## Abstract

Sleep disorders and stroke are intricately linked through a complex, bidirectional relationship. Sleep disturbances such as obstructive sleep apnea (OSA), insomnia, and restless legs syndrome (RLS) not only increase the risk of stroke but also frequently emerge as consequences of cerebrovascular events. OSA, in particular, is associated with a two- to three-fold increased risk of incident stroke, primarily through mechanisms involving intermittent hypoxia, systemic inflammation, endothelial dysfunction, and autonomic dysregulation. Conversely, stroke can disrupt sleep architecture and trigger or exacerbate sleep disorders, including insomnia, hypersomnia, circadian rhythm disturbances, and breathing-related sleep disorders. These post-stroke sleep disturbances are common and significantly impair rehabilitation, cognitive recovery, and quality of life, yet they remain underdiagnosed and undertreated. Early identification and management of sleep disorders in stroke patients are essential to optimize recovery and reduce the risk of recurrence. Therapeutic strategies include lifestyle modifications, pharmacological treatments, medical devices such as continuous positive airway pressure (CPAP), and emerging alternatives for CPAP-intolerant individuals. Despite growing awareness, significant knowledge gaps persist, particularly regarding non-OSA sleep disorders and their impact on stroke outcomes. Improved diagnostic tools, broader screening protocols, and greater integration of sleep assessments into stroke care are urgently needed. This narrative review synthesizes current evidence on the interplay between sleep and stroke, emphasizing the importance of personalized, multidisciplinary approaches to diagnosis and treatment. Advancing research in this field holds promise for reducing the global burden of stroke and improving long-term outcomes through targeted sleep interventions.

## 1. Introduction and Historical Background

Cryptogenic strokes, defined as ischemic cerebrovascular events of unknown etiology, are frequently associated with SDB. SDB encompasses a spectrum of conditions characterized by abnormal respiratory patterns and ventilatory disturbances, including hypoxemia and hypercapnia. These disorders include OSA, CSA, sleep-related hypoventilation, CSB, RLS, insomnia, REM sleep behavior disorder, and PLMS. Approximately 50–70% of stroke patients exhibit suspected SDB, with a higher prevalence observed among individuals experiencing recurrent cerebrovascular events compared to those with a single ischemic episode [1]. The relationship between sleep and stroke is bidirectional as follows: acute stroke can precipitate the onset of SDB, while pre-existing sleep disturbances significantly elevate the risk of cerebrovascular events. Notably, patients with a history of OSA prior to stroke have been shown to experience prolonged hospital stays—by an average of 14 days—and greater functional impairment compared to those without prior OSA [2].

Effective treatment of sleep disorders, particularly OSA, is critical, as OSA not only contributes to the initial occurrence of stroke but is also associated with increased risk of recurrent stroke and poorer post-stroke recovery [3]. Management of OSA has been shown to enhance functional outcomes, including reductions in post-stroke depression, improvements in daily living activities, and better cognitive performance, such as attention and concentration [4]. Epidemiological studies have consistently demonstrated that SDB is linked to a two- to three-fold increased risk of incident stroke. However, disentangling the causal relationship between OSA and stroke remains challenging due to overlapping demographic and clinical risk factors—such as age, sex, obesity, hypertension, hyperlipidemia, and diabetes mellitus—that are common to both conditions. The SHHS, a large prospective cohort study, followed approximately 5000 individuals over eight years from the time of OSA diagnosis, assessing the long-term cardiovascular consequences, including the incidence of ischemic stroke [4].

This narrative review aims to provide an overview of the sleep disorders that increase the risk of stroke, including their pathophysiology, available screening tools, and management. It also seeks to examine the development of sleep disorders as a result of stroke (Supplementary Material Section S1—Methodology).

## 2. Sleep Disorder as a Risk Factor for Stroke

Sleep disorders have been recognized as significant risk factors for stroke. Studies by Munoz et al. [5] and Hornyak et al. [6] revealed that there is a close correlation between SDB, particularly OSA, and an increased incidence of stroke. In agreement, Plomaritis et al. acknowledged the negative impacts of insomnia and other sleep disruption patterns on cerebrovascular health [7]. Franklin et al. (2015) found that poor quality of sleep, as well as disturbances in sleep patterns, were independent predictors of the occurrence of stroke [8]. Ma et al. also reported similar findings that increased sleep disturbance and poor sleep quality were independent risk factors for stroke [9]. In 2018, Dauvilliers et al. further highlighted bidirectionality, with sleep disorders not just increasing the risk of stroke but worsening post-stroke outcomes [10]. Other similar studies by Morin et al. [11] and Leng et al. [12] demonstrated that the proper management of sleep disorders could have the ability to prevent stroke risk. Treatment of OSA significantly reduces stroke incidence, emphasizing the importance of early diagnosis. Early identification and management of sleep disorders can mitigate their role as independent stroke risk factors and contribute to a more comprehensive prevention strategy [13]. The incidence of different sleep disorders and the risk related to stroke are summarized in Table S1 [5,6,7,8,9,10,11,12,13,14,15].

### 2.1. Sleep, Ambulatory Blood Pressure (ABP), and Risk of Stroke

Elevated daytime maximum ABP is strongly associated with increased stroke risk, particularly in individuals with heightened arterial stiffness. Subjects in the highest quartile of daytime maximum BP exhibit a significantly greater incidence of stroke compared to those in lower quartiles [16]. In older adults, ABP monitoring provides more reliable stroke risk prediction than office-based measurements. Among ABP parameters, diastolic pressure is a stronger predictor than systolic pressure due to its closer association with vascular resistance and perfusion in small cerebral vessels. Additionally, 24 h systolic BP profiles are linked to short-term stroke recurrence, and comprehensive ABP profiling may aid in early detection of recurrent events [17].

Nocturnal hypertension, defined by home or ambulatory SBP ≥120 mm Hg during sleep, is associated with increased cardiovascular risk, including a 2.65-fold higher risk of stroke [18]. This risk is further elevated in atrial fibrillation patients with a reverse-dipper BP pattern and in those with reduced nocturnal BP dips [19], such as patients on HeartMate II support [20]. Incorporating sleep duration and nocturnal BP into cardiovascular risk models significantly improves stroke prediction (C-statistic: 0.795). These findings underscore the importance of managing nocturnal hypertension and maintaining optimal sleep duration to reduce stroke risk [21]. Additionally, distinct circadian BP patterns observed in AIS may reflect sympathetic nervous system activity and stress responses, warranting further investigation into their clinical relevance [22].

### 2.2. Obstructive Sleep Apnea (OSA)

OSA is a prevalent sleep-related breathing disorder marked by recurrent upper airway obstruction during sleep, leading to oxygen desaturation, sleep fragmentation, and heightened sympathetic activity [23]. Meta-analyses have shown that OSA is associated with a twofold increased risk of stroke (RR ≈ 2.0) [24]. Case–control studies further support this link, showing higher rates of cardioembolic stroke in patients with OSA and a 50% increase in recurrent stroke or TIA among those with SDB [25]. A summary of these findings is presented in Table S2 [5,26,27,28,29,30,31,32,33,34].

The pathogenesis of OSA is multifactorial, involving autonomic, hemodynamic, and biochemical dysregulation [35]. Intermittent hypoxia during apneic episodes activates the sympathetic nervous system, leading to elevated vascular resistance, systemic inflammation, and increased levels of catecholamines, angiotensin II, and endothelin-1. These changes contribute to the development of hypertension, cardiovascular disease, and heightened stroke risk [36].

#### 2.2.1. Incidence of Stroke and Obstructive Sleep Apnea

OSA is both a prevalent comorbidity and an independent risk factor in stroke patients, significantly influencing outcomes and mortality. In one study, only 82.7% of stroke patients with OSA survived one-year post-event, with mortality significantly higher than in those without OSA (*p* = 0.01). Male patients with OSA had particularly poor survival rates (*p* = 0.004). Stroke patients with OSA also more frequently present with cardiovascular risk factors, hypertension, and elevated body mass index—factors associated with worse prognosis and increased mortality [37].

A 2019 meta-analysis reported a pooled prevalence of OSA in stroke patients at 71%, based on an AHI >5 events/hour [38]. A subsequent meta-analysis of 75 studies involving 8670 stroke patients confirmed this high prevalence and further stratified it by stroke type and location as follows: OSA was more common in hemorrhagic than ischemic stroke [82.7% vs. 67.5%, *p* = 0.098], and it was slightly more prevalent in supratentorial than infratentorial strokes [64.4% vs. 56.5%, *p* = 0.171]. These findings reinforce the high burden of OSA in stroke populations [39]. In contrast, CSA was found to be uncommon among patients with AIS [40].

The relationship between sleep disturbances and stroke is bidirectional: sleep disorders increase stroke risk, while stroke can induce or worsen sleep disturbances. Mechanistically, stroke-related sleep disruptions are linked to functional brain changes, neurotransmitter imbalances, and inflammation [41]. In OSA, factors such as non-dipping blood pressure, hypoxemia, sympathetic activation, and impaired cerebral hemodynamics contribute to stroke risk, with CPAP therapy proposed to improve outcomes [42]. OSA-specific mechanisms include arrhythmia, paradoxical embolism, atherosclerosis, metabolic dysfunction, and vibratory injury from snoring [43]. Other sleep disorders—such as insomnia, abnormal sleep duration, and movement disorders—are also associated with stroke through pathways involving inflammation, oxidative stress, autonomic dysregulation, and endothelial dysfunction [44].

#### 2.2.2. Sleep Duration and Stroke

Both short (<7 h) and long (≥9 h) sleep durations are associated with increased risk of stroke and poorer post-stroke outcomes. Long sleep is linked to higher rates of ischemic stroke and intracerebral hemorrhage, while short sleep is particularly associated with intracerebral hemorrhage [45]. Individuals sleeping less than 6 h face nearly double the stroke risk (OR: 1.97), and those with sleep disorders also show elevated risk (OR: 1.71) [46]. Stroke itself contributes to sleep disturbances, including frequent awakenings and early morning arousals, which are associated with increased risk of post-stroke depression, anxiety, and cognitive impairment [47]. Both inadequate and excessive sleep have been identified as risk factors for cognitive decline at multiple post-stroke intervals [48]. These associations are especially pronounced in women, middle-aged adults, and individuals with metabolic syndrome [49]. Evidence supports maintaining a sleep duration of 7–9 h as optimal for stroke prevention and recovery [50]. Further analysis in different cohorts found that abnormal sleep duration was related to increased stroke odds, which also encouraged maintaining a balanced sleep schedule [51].

#### 2.2.3. Sleep Duration and Special Populations

Sleep duration varies significantly across racial and ethnic groups, influenced by socioeconomic and occupational factors. Studies have shown that African and Asian Americans tend to have delayed sleep–wake patterns compared to non-Hispanic White people, possibly due to circadian rhythm differences shaped by environmental and social conditions [52]. Disparities in sleep disorders across populations further highlight the need for equitable healthcare strategies [53]. Genetic studies have identified 84 loci associated with short sleep and one with long sleep duration across diverse ancestries, suggesting a shared genetic basis for sleep traits [54]. Additionally, rare genetic syndromes, such as Down and Prader–Willi [55], and structural abnormalities, like mucopolysaccharide accumulation in the airways, contribute to sleep-disordered breathing [56].

Sleep duration disparities are evident across racial and ethnic groups, influenced by socioeconomic and environmental factors. African Americans and Native Hawaiians/Pacific Islanders are more likely than Hispanics to report short sleep durations, while African immigrants and refugees, as well as African Americans, show less interest in improving sleep despite associated symptoms like depression and stress [57]. Native Hawaiians and Pacific Islanders also report significantly shorter sleep compared to White people, whereas Asians are less likely to experience unhealthy sleep durations. Among youth, short sleep ranges from 31.2% in teens to 40.3% in infants, with consistent bedtime routines linked to adequate sleep [58]. Socioeconomic status and urban living have also been identified as contributors to reduced sleep duration [59].

Sleep disorders exhibit diverse etiologies across populations and comorbid conditions. In epilepsy, higher rates of RLS and sleep bruxism have been observed, while snoring and poor sleep hygiene are less frequently reported [60]. Insomnia is more prevalent among individuals with personality disorders and PTSD, and sleep-related breathing disorders are common in those with substance use disorders and ADHD. Parasomnias are most frequent in patients with PTSD, followed by those with substance use and cluster B personality disorders [61]. In migrant populations, sleep disturbances may stem from unique risk factors, traumatic experiences, and lifestyle changes related to integration, including altered dietary habits and work schedules, which can contribute to conditions like OSA and insomnia [62].

### 2.3. Insomnia

Insomnia is defined by difficulty initiating or maintaining sleep, with awakenings lasting over 30 min occurring at least three times per week. It is classified as short-term if lasting less than three months and chronic if persisting longer [63]. Nearly 50% of stroke patients experience insomnia in the early post-stroke period, with 33% reporting it as a new symptom and the remainder as a recurrence [64,65]. Approximately 30% of stroke survivors report insomnia, which is often associated with elevated anxiety levels and greater functional disability [66].

In a pediatric population study of 16,609 individuals aged 18 or younger, sleep-disordered breathing (SDB) was the most common diagnosis, accounting for 11.2% of cases, followed by nocturnal enuresis (1.2%) and insomnia (1%). Sleep disorders were significantly more prevalent among those with multiple chronic medical conditions compared to those with a single condition (19.7% vs. 5.8%, *p* < 0.001). Additionally, insomnia prevalence was lower among Hispanic (1.2%) and African American (0.8%) children and adolescents compared to Caucasians (3.5%) (*p* < 0.001) [67]. For detailed studies on insomnia and stroke, refer to Table S3 [68,69,70,71,72,73,74,75,76].

Using EEG, Sterr et al. observed that post-stroke patients exhibited increased sleep latency and reduced sleep efficiency, characterized by prolonged wakefulness after sleep onset and a higher proportion of time spent awake. Despite similar total sleep durations across groups, patients showed greater difficulty initiating sleep during the day and demonstrated increased error rates in vigilance tasks [77].

Zhang et al. investigated potential biochemical markers of post-stroke insomnia (PSI) but found no significant associations with Cell Counting Kit-8 (CCK-8), substance P (SP), or serotonin (5-HT), suggesting the need to explore additional neurotransmitters [48]. In a related study, they assessed serum levels of glutamine (GLN), glutamate (GLU), and gamma-aminobutyric acid (GABA), finding no significant differences between PSI and post-stroke depression groups. However, both groups exhibited significantly lower levels of these neurotransmitters compared to healthy controls, indicating a possible shared neurochemical disruption [48].

Wang et al. demonstrated that post-stroke insomnia is linked to hyperactivation of the DMN and heightened audiovisual sensitivity, disrupting sleep-regulating brain regions [78]. Mayer et al. further identified post-stroke insomnia as a disorder of the brain’s sleep–wake centers, often accompanied by cognitive and emotional impairments, and they found it responds well to cognitive behavioral therapy, antidepressants, melatonin, and GABA agonists [79].

Stroke risk is elevated in individuals under 50 with comorbidities, though it can be reduced through public education, early detection, and effective management [80]. Post-stroke survivors exhibit significantly higher rates of insomnia and related symptoms compared to the general population [65]. Insomnia following stroke is associated with a greater risk of long-term cognitive impairment. Fleming et al. reported that dCBT significantly improves sleep quality, insomnia symptoms, and mood in stroke patients [81]. Additionally, Huang et al. found that combining electroacupuncture with Suanzaoren decoction is more effective and safer for treating post-stroke insomnia than either treatment alone [82].

The severity of insomnia does not significantly influence motor recovery in ischemic stroke patients, where age and NIHSS scores are more predictive of clinical outcomes. However, insomnia remains a critical factor in post-stroke recovery, contributing to anxiety, cognitive decline, and reduced quality of life. Chronic insomnia may be an independent risk factor for stroke, though more standardized studies are needed to confirm this association [83]. The relationship between stroke, insomnia, and conditions like RLS and PLMS is still under investigation. Evidence suggests that supratentorial strokes may disrupt cortical inhibition, potentially triggering PLMS contralateral to the lesion [84]. RLS has also been linked to increased cardiovascular and cerebrovascular risk, including stroke. Digital cognitive behavioral therapy and acupuncture-based treatments have shown promising results in improving sleep and mood outcomes in post-stroke patients [85].

### 2.4. Restless Legs Syndrome (RLS)

The prevalence of RLS varies geographically, affecting 1–3% of individuals in Asia and 5–13% in Europe and North America [14]. Women are 30–50% more likely to develop RLS than men, with incidence increasing with age, typically beginning in middle age [14]. RLS has been associated with elevated cardiovascular risk as follows: a study of 4000 men found a 2.5-fold increased likelihood of heart problems and a similar link to hypertension [25]. Also, men aged 55–69 reported a 66% higher risk of ischemic stroke in those with RLS. Additionally, patients with end-stage renal disease and RLS had a 2.5-fold increased risk of stroke compared to those without RLS [25]. To further understand the relationship between RLS and stroke, read Table S4 [86,87,88].

RLS is a multifactorial neurological disorder influenced by the following three primary mechanisms: iron deficiency, dopaminergic dysfunction, and genetic predisposition. Iron deficiency in brain regions such as the substantia nigra, putamen, caudate, and thalamus contributes to neuronal hypoxia and demyelination. Dopaminergic abnormalities include elevated dopamine synthesis—evidenced by increased 3-orthomethyl dopamine and reduced fluoro-L-dopa uptake—as well as decreased dopamine transporter availability and D2 receptor density. Genetically, variants in loci such as BTBD9, PTPRD, MAP2K5/SKOR1, MEIS1, and TOX3 have been strongly associated with RLS susceptibility [89].

### 2.5. REM Sleep Behavior Disorder

RBD is characterized by dream enactment behaviors, such as motor activity or vocalizations [90], due to the loss of normal muscle atonia during REM sleep, as confirmed by polysomnography [91]. It affects approximately 11% of stroke survivors and is considered a potential early marker of neurodegenerative disease [1]. Over 90% of individuals with iRBD eventually develop synucleinopathies, including Parkinson’s disease, dementia with Lewy bodies, or, less commonly, multiple system atrophy, often more than 15 years after symptom onset [92].

Patients with iRBD often exhibit autonomic dysfunction, such as a non-dipping blood pressure profile, which may contribute to an elevated CVD risk [93]. Sleep-related movement disorders, including RBD, are associated with a significantly increased risk of stroke, with an adjusted hazard ratio of 2.29 (95% CI: 1.42–3.80) [94]. Ma et al. further confirmed that RBD increases the risk of both ischemic and hemorrhagic strokes [95]. While brainstem lesions are implicated in RBD, the disorder is primarily linked to neurodegeneration of glutamatergic neurons in the pons and GABAergic neurons in the medulla [96].

Acute ischemic stroke (AIS) is often followed by a marked reduction in REM sleep, a change that correlates with poorer clinical outcomes. Notably, prolonged REM sleep latency has been identified as an independent predictor of stroke progression. While these findings suggest a potential role of REM sleep in stroke recovery, the underlying mechanisms remain unclear [97].

### 2.6. Periodic Limb Movements of Sleep

According to the American Academy of Sleep Medicine, PLMS are defined as repetitive limb movements occurring at a frequency of at least 15 per hour in adults [90]. While commonly associated with RLS, PLMS can also occur in other sleep disorders, medical conditions, or even in healthy individuals [6]. RLS has been linked to increased risks of diabetes, hypertension, obesity [98], and a higher prevalence of coronary artery and cardiovascular disease—particularly in individuals with more frequent or severe symptoms [99]. However, current evidence on RLS as a predictor of cardiovascular events and all-cause mortality remains limited and inconclusive [100]. As Bassetti et al. highlight, further research is needed to clarify the connections between insomnia, RLS/PLMS, and stroke [84].

Del Brutto et al. found no independent association between the periodic limb movement index and neuroimaging markers of cSVD in older adults without a history of stroke [101]. However, genetic studies have linked PLMS to increased risks of stroke, RLS, and insomnia [102]. More recently, Plomaritis et al. reported that severe PLMS occurs in approximately 76% of patients with acute stroke and is associated with poorer outcomes. These findings underscore the importance of early detection and management of PLMS in stroke care [7].

PLMS have been linked to a low respiratory arousal threshold, suggesting a potential connection with the non-anatomical arousal mechanisms seen in OSA [103]. PLMS also appears to accelerate the progression of WMH, serving as a predictor of cSVD burden [104]. Additionally, PLMS is associated with an increased risk of hypertension [105]. While some studies, such as that by Malkiewicz et al., report elevated parasympathetic activity during PLMS episodes, this autonomic co-activation may contribute to fatal cardiac events [106].

### 2.7. Shift Work

Recent studies have shown that working 48–54 h or more per week is associated with an increased risk of stroke [107]. Moderate-grade evidence supports a direct relationship between long working hours and stroke incidence [108]. Theorell et al. suggest that the elevated cardiovascular risk observed in shift workers may be due to acute sympathetic nervous system activation, which disrupts the excretion of catecholamines and alters levels of cholesterol, uric acid, glucose, and potassium [109].

Shift work and extended work hours are associated with both acute and chronic health risks, including metabolic syndrome and certain cancers, due to shared biological pathways [110]. Shift workers exhibit elevated levels of inflammatory markers compared to day workers [111], suggesting a biological link between work schedule demands and chronic disease [112]. Night shift work, in particular, is associated with increased systemic inflammation, notably involving IL-6 [113] and pentraxin 3 [114]. Additionally, shift work has been linked to the early onset of AF, particularly in individuals under 40 years of age over a 10-year follow-up period [115].

In an animal study, Ramsey et al. demonstrated that chronic disruption of the intrinsic sleep–wake cycle—mimicking shift work—impairs stroke recovery, likely through heightened activation of the brain’s immune response. These findings suggest that the negative health effects of shift work may be mitigated through interventions targeting work schedules or inflammatory pathways [116].

Both short- and long-term exposure to night shift work increases the risk of AF, independent of genetic predisposition, and it is also associated with a higher incidence of CHD. However, night shift work does not appear to significantly affect the risk of stroke or heart failure. Skogstad et al. reported that prolonged shift work is linked to preclinical cardiovascular changes, such as increased carotid intima-media thickness and elevated C-reactive protein, indicating a higher risk for CHD and stroke [117]. Additionally, the MTNR1B rs10830963 polymorphism may mediate the relationship between night shift work and stroke risk [118]. Female shift workers are particularly vulnerable to psychological issues, especially depression [119], which itself is associated with a significantly increased risk of stroke (RR = 1.40, 95% CI: 1.27–1.53, *p* < 0.0001) [120].

Shift workers, particularly those with long-term night shift schedules, have higher odds of CMM related to hypertension compared to day workers [121]. Among female, non-Hispanic White nurses, rotating night shift work was independently associated with an increased risk of ischemic stroke, with a 4% rise in risk for every five years of shift work [122]. While some studies, such as Hermansson’s, did not find a significant difference in stroke risk between shift and day workers [123], more recent evidence indicates a modest but significant increase in stroke risk associated with night shift work (HR 1.13; 95% CI: 1.00–1.28) [118]. Overall, the cumulative data suggest that night shifts and long work hours elevate stroke risk and worsen outcomes, primarily through circadian rhythm disruption and heightened inflammatory responses. Interventions targeting shift patterns or inflammatory pathways may help mitigate these risks.

## 3. Sleep Disorders as a Consequence of Stroke

PSSD encompasses a range of sleep disturbances following a cerebrovascular accident or TIA, including CSA, OSA, insomnia, EDS, NSD, and RLS. These disorders arise from stroke-related changes in neural regulation of sleep, breathing, and circadian rhythms, and they may also be influenced by psychosocial stress, hormonal imbalances, oxidative stress, and immune activation. A meta-analysis of 64 studies reported insomnia prevalence at 40.7% in the acute phase (<1 month), 42.6% in the subacute phase (1–3 months), and 35.9% in the chronic phase (>3 months) [124]. RLS was found in 10.4% of patients during the acute phase and 13.7% in the chronic phase [124]. To further understand the incidence of sleep disorders and post-stroke risk, read Table S5 [1,38,64,125,126,127,128,129,130,131,132,133,134].

### 3.1. Sleep-Related Breathing Disorders

OSA and CSA are classified under SDB and affect up to 50% of post-stroke patients [135,136]. OSA results from mechanical collapse of the upper airway during sleep, influenced by factors such as age, obesity, male sex, and supine sleeping position (Table S6 [137,138,139,140,141,142]). Reduced tone in pharyngeal dilator muscles like the genioglossus during sleep further contributes to airway narrowing and hypopnea-apnea episodes [136]. These events can lead to hypoventilation, carbon dioxide retention, respiratory acidosis, and sympathetic arousal, often causing sleep fragmentation. CSA, on the other hand, is linked to dysfunction in the brainstem respiratory centers—specifically the dorsal and ventral respiratory groups, the pre-Bötzinger complex, and the pontine pneumotaxic and apneustic centers. Lesions in these areas, common in stroke, can disrupt respiratory rhythm and lead to CSA.

Research on the relationship between stroke lesion location and the incidence of SDB has yielded mixed results. Some case–controlled studies suggest that infratentorial lesions—those affecting the brainstem and cerebellum—are associated with higher AHI scores [135]. Conversely, other studies have found that total anterior circulation strokes, which involve large infarcts in the territories of the anterior and middle cerebral arteries, show the strongest correlation with SDB [143]. A prevailing hypothesis for cortical SDB posits that increased intracranial pressure following stroke may compress respiratory-related neurons, contributing to disordered breathing during sleep.

### 3.2. Circadian Rhythm Disorders

Stroke incidence follows a bimodal distribution, with peaks occurring in the early morning (6:00 a.m. to 12:00 p.m.) and at night [144]. Epidemiological data show that the morning peak coincides with elevated blood pressure and sympathetic nervous system activity [145]. This increased morning risk appears to be independent of stroke subtype and traditional risk factors such as diabetes and hyperlipidemia. Wake-up strokes present a clinical challenge due to the uncertainty of symptom onset, complicating eligibility for time-sensitive thrombolytic therapy (e.g., tPA), which is recommended within 4.5 h of ischemic stroke onset [146]. These patterns underscore a temporal relationship between stroke occurrence and circadian rhythm disturbances.

The sleep–wake cycle is regulated by several key brain regions, including the pineal gland, the SCN, the sleep-promoting VLPO, and multiple wake-promoting nuclei such as the tuberomammillary nucleus, lateral hypothalamus, locus coeruleus, dorsal raphe, laterodorsal tegmental nucleus, and pedunculopontine tegmental nucleus. Preliminary studies have shown that stroke patients often experience a delayed melatonin rhythm during the acute phase, indicating circadian disruption [147]. Additionally, a 2004 study found that the typical 10% nocturnal blood pressure dip—known as diurnal dipping—was absent in acute stroke patients, further supporting the role of circadian dysregulation in post-stroke physiology [148].

### 3.3. Post-Stroke Insomnia

Insomnia, defined as difficulty initiating or maintaining sleep, can present acutely or chronically. Population-based studies estimate that approximately 30% of individuals experience insomnia symptoms [128]. However, when applying stricter diagnostic criteria that include daytime impairment or distress—as outlined by the NIH State-of-the-Science Conference in 2005—the prevalence drops to around 10% [129]. Among stroke patients, 20–56% report insomnia, with 18% experiencing new-onset insomnia following the stroke [130].

The location of a stroke plays a critical role in the development of insomnia due to the involvement of multiple brain regions in sleep–wake regulation. Key structures include the reticular formation in the brainstem, thalamus, hypothalamus, and pineal gland. Strokes affecting the paramedian thalamus can impair the thalamic reticular system, disrupting sleep spindle generation [130]. Focal lesions may also alter neurotransmitter dynamics: the VLPO promotes sleep via GABA and galanin; the locus coeruleus and dorsal raphe nucleus promote wakefulness through norepinephrine and serotonin, respectively; the basal forebrain supports wakefulness and REM sleep via acetylcholine; and the pineal gland regulates circadian rhythms through melatonin secretion. Damage to any of these regions can disrupt sleep architecture, contributing to post-stroke insomnia.

In addition to focal brain lesions, psychosocial factors significantly contribute to post-stroke insomnia. Many stroke survivors experience heightened anxiety about their health and recovery, which can elevate sympathetic nervous system activity. This increased sympathetic tone disrupts normal sleep physiology, leading to reduced sleep quality and difficulty initiating or maintaining sleep.

### 3.4. Post-Stroke Hypersomnia

EDS is commonly reported during the acute phase following a stroke [149,150], with prevalence estimates ranging from 18% to 72%, depending on the assessment method and stroke subtype [151,152,153]. Despite this variability, approximately 34% of stroke survivors experience persistent EDS lasting at least six months post-stroke. EDS can significantly impair recovery and quality of life [154,155]. Hypersomnia, characterized by prolonged sleep during both day and night, reduced activity, and negative impacts on physical and psychological health [156], may also occur post-stroke and can co-occur with other neurological disorders [157].

Hypersomnia following stroke is often associated with lesions in the thalamus, mesencephalon, or pontine tegmental reticular formation [158]. More recently, lesions near the paraventricular nucleus (PVN) of the hypothalamus have also been implicated in newly identified cases of post-stroke hypersomnia, which often improve with recovery [78]. Chen et al. highlighted the central role of PVN dysfunction in hypersomnolence disorder. Specifically, glutamatergic neurons in the PVH^vglut2^, including subpopulations such as PVH^OT^, PVH^PDYN^, and PVH^CRH^, are critical for maintaining wakefulness [159].

Post-stroke hypersomnia can result from damage to the ventral lower ARAS [160], a key structure in maintaining wakefulness [161]. It may also occur as a rare manifestation of bilateral thalamic infarcts, which can have severe long-term consequences if not promptly diagnosed and treated [162]. Saida et al. emphasized the importance of considering post-stroke hypersomnia in differential diagnoses, especially in cases of hypersomnolence at work [163]. Cognitive-communication impairments have also been linked to sleep disturbances following brain trauma, with evidence suggesting that pharmacological improvement of sleep can enhance cognitive and communication outcomes [164]. Harris et al. noted that hypersomnia and apathy significantly reduce participation in rehabilitation, leading to poorer recovery [165]. Osman et al. reported a case of hypersomnia following bilateral thalamic stroke, highlighting the diagnostic challenges and the value of early diffusion-weighted MRI in timely detection and intervention [166].

### 3.5. Sleep-Related Movement Disorders After Stroke

RLS is characterized by an uncontrollable urge to move the legs, often accompanied by crawling, tingling, or burning sensations that are relieved by movement. In contrast, periodic limb movement syndrome (PLMS) involves repetitive, involuntary limb movements during sleep. Post-stroke RLS has been reported in 2.3–15.1% of patients, while post-stroke PLMS is considerably rarer [167]. To date, only 30 cases of post-stroke primary RLS and PLMS have been documented. Focal ischemic lesions in these cases were most commonly located in the pons [168]. Post-stroke RLS typically presents bilaterally and is associated with lesions in the corona radiata and basal ganglia, whereas post-stroke PLMS is usually unilateral and linked to lesions in the pontine base and tegmentum [168]. For a further understanding of sleep-related movement disorders after stroke, read Table S7 [124,168,169,170,171,172,173,174,175].

### 3.6. Sleep Disorders and Post-Stroke Depression

PSD affects approximately one-third of stroke survivors [176]. Its pathophysiology is multifactorial, involving both psychosocial and biological mechanisms. Functional and cognitive impairments post-stroke are associated with increased depression risk [177], suggesting a psychosocial component [176]. Biologically, PSD has been linked to dysregulation in neurotransmitters, hormones, oxidative stress, and immune responses. Evidence supports a direct neurobiological basis [178], as PSD can occur independently of physical disability and is observed in patients with anosognosia [179]. While lesion location has shown inconsistent associations with PSD, epigenetic factors—such as methylation of the SLC6A4 and BDNF—have been implicated [176]. Additionally, HPA axis overactivation during acute illness may contribute via elevated cytokine and CRH levels [180]. For a summary of all the post-stroke sleep disorders, read Figure S1.

## 4. Pathophysiology of Sleep Disorders and Stroke

Sleep disorders and stroke are interlinked through a bidirectional relationship, where each condition can exacerbate the other [181]. Shared pathophysiological mechanisms—particularly intermittent hypoxia, systemic inflammation, and autonomic dysfunction—underlie this connection [182]. Disrupted sleep architecture, common in many sleep disorders, contributes to cerebrovascular vulnerability by promoting endothelial dysfunction, blood pressure variability, and prothrombotic states. These changes not only elevate stroke risk but may also influence stroke severity and recovery [183]. Understanding these overlapping mechanisms is essential for improving stroke prevention, management, and patient outcomes (Figure S2).

Hypoxia is a central mechanism linking sleep disorders and stroke, contributing significantly to the pathophysiology of both [184]. In stroke, vascular occlusion leads to cerebral ischemia, depriving brain tissue of oxygen and nutrients, resulting in neuronal injury [185]. In SDB—particularly OSA—recurrent episodes of upper airway obstruction cause intermittent hypoxia, hypercapnia, and sleep fragmentation [186]. These apneic events trigger oxidative stress, sympathetic activation, and systemic inflammation [187]. Chronic intermittent hypoxia, a hallmark of OSA, promotes endothelial dysfunction and increases stroke risk [188]. Furthermore, reperfusion injury following ischemia introduces ROS, exacerbating cellular damage.

In OSA, repeated episodes of hypoxia and reoxygenation induce oxidative stress, damaging the vascular endothelium and disrupting blood flow regulation [159]. This promotes atherosclerosis, particularly in cerebral vessels [189], increasing stroke risk [190]. Additionally, chronic hypoxia in OSA elevates blood viscosity and alters platelet function, creating a hypercoagulable state that further heightens the likelihood of stroke [184].

Beyond vascular effects, OSA impairs cardiac function, linking sleep disruption to stroke risk [173]. Repeated hypoxia-reoxygenation strains the heart, promoting arrhythmias like atrial fibrillation [191], which increases stroke risk [192] through clot formation and cerebral embolism [193]. Even without overt heart disease, OSA can alter left ventricular structure and function [194]. Additionally, OSA disrupts cerebral autoregulation, reducing the brain’s ability to maintain stable blood flow during blood pressure fluctuations [195,196]. This impaired response worsens ischemic damage during stroke [197,198]. Thus, OSA contributes to stroke through both cardiovascular dysfunction and impaired cerebral hemodynamics.

Inflammation, a key process in tissue repair and immune defense, also plays a significant role in the shared pathophysiology of stroke and sleep disorders [1,199].

While inflammation initially aids in clearing cellular debris and initiating repair after stroke [200], persistent inflammation in the ischemic brain exacerbates neuronal injury and hinders recovery [201]. In OSA, systemic inflammation—likely driven by oxidative stress from intermittent hypoxia [199]—contributes to atherosclerosis [199]. Elevated inflammatory markers in OSA accelerate plaque formation in cerebral arteries, compounding stroke risk beyond that caused by hypoxia or impaired cerebral autoregulation [202].

Autonomic dysfunction is a key link between sleep disorders and stroke, often exacerbating both [203,204]. Sleep-disordered breathing (SDB), particularly OSA, activates the sympathetic nervous system in response to hypoxemia during apneic events [84,186], causing transient spikes in blood pressure and heart rate [204]. Chronic sympathetic activation contributes to sustained hypertension and blunts the normal nocturnal BP dip, increasing stroke risk [205]. Conversely, stroke—especially in the brainstem—can impair autonomic regulation, worsening preexisting sleep disturbances like SDB [206]. Addressing this bidirectional relationship offers a critical opportunity to improve outcomes in affected patients [207].

Integrating validated screening tools like STOP-BANG, ESS, and the newer SLEEP Inventory into routine stroke care can enhance recognition of sleep disorders and guide targeted interventions [137]. In acute stroke settings, the DOC screen offers a rapid, holistic assessment of mood, cognition, and sleep disturbances [208]. Objective sleep studies further confirm diagnoses and inform personalized treatment [190]. Managing OSA with CPAP has shown benefits, and strategies like telehealth support may improve adherence, reduce recurrent stroke risk, and aid recovery [209,210]. This integrated approach reframes sleep disorders not just as risk factors but as modifiable contributors to stroke outcomes.

### 4.1. Normal Sleep and Cerebral Hemodynamics

Sleep is a periodic brain state marked by reduced activity and consciousness, regulated by the two-process model: Process C (circadian rhythm) governed by the SCN, and Process S (homeostatic drive) involving sleep-promoting substances like adenosine [211]. Sleep cycles through four NREM stages and one REM stage, with NREM dominated by parasympathetic activity and reduced cerebral perfusion (5–28%) [211], while REM is driven by sympathetic activity and increased perfusion (4–41%) [211]. Central chemoreceptors respond to pCO_2_, and these perfusion shifts influence breathing patterns across sleep stages.

Sleep is a complex physiological process essential for brain health and cognitive function [212], comprising distinct stages with specific roles in neural restoration and plasticity [212]. Disruptions—such as insufficient duration, fragmentation, or sleep disorders—can impair cerebral hemodynamics, contributing to neurological deficits. Studies, including those by Somers et al., highlight the strong link between sleep disturbances and cognitive decline. Sleep is broadly divided into NREM (stages N1, N2, and N3) and REM, each with unique electrophysiological and physiological characteristics [213].

As sleep deepens from stage 1 to stage 3, EEG frequency progressively slows, with N3 (deep sleep) dominated by delta waves (1–4 Hz) [214], reflecting reduced neuronal activity and metabolic demand [215]. In contrast, REM sleep features vivid dreams, muscle atonia [216], and increased theta (4–10 Hz) and gamma (35–58 Hz) activity in the cortex and hippocampus, indicating heightened neuronal activity and brain function [217,218]. REM sleep behavior disorder disrupts this normal REM pattern.

Sleep is accompanied by characteristic physiological changes: heart rate and blood pressure decline during NREM sleep, reaching their lowest in deep sleep (N3), while REM sleep shows marked fluctuations, often exceeding waking levels [219]. These changes reflect autonomic nervous system activity and influence cerebral hemodynamics [219]. Adequate sleep—7–9 h for young and middle-aged adults—is essential for cognitive performance, immune function, and hormonal balance. Chronic sleep deprivation or disruption can impair brain function and cognition [220].

Intrinsic cerebral hemodynamics—the regulation of blood flow within the brain—is essential for maintaining brain health. NVC, also known as functional hyperemia, ensures that active brain regions receive adequate blood supply [221]. Triggered by increased neuronal activity, NVC involves local arteriole vasodilation to deliver oxygen and nutrients, supporting metabolic demands and cognitive function [222].

Cerebral autoregulation maintains stable CBF despite fluctuations in systemic blood pressure by adjusting the diameter of cerebral arterioles [223]. Impairment of this mechanism can lead to hypoperfusion or hyperperfusion, increasing the risk of ischemic injury or damage to sensitive brain regions. CVR reflects the brain’s ability to dilate vessels in response to stimuli like hypercapnia or hypoxia, serving as an important indicator of vascular health [224].

Several techniques are used to assess cerebral hemodynamics. Transcranial Doppler ultrasonography non-invasively measures blood flow velocity in major cerebral arteries. fMRI detects BOLD signals, reflecting neuronal activity via deoxyhemoglobin changes [225]. fNIRS tracks hemoglobin concentration shifts to assess cortical blood volume and oxygenation [226]. DVA measures retinal microvessel diameter, offering insights into cerebrovascular reactivity and microvascular health [227].

Neuropsychological tests like CANTAB and n-back consistently show that sleep deprivation impairs reaction time, sustained attention, and working memory [228,229,230]. Transcranial Doppler studies reveal altered CBF patterns in sleep-deprived individuals, suggesting disrupted regulatory mechanisms [231]. NIRS also shows reduced hemodynamic responses in the prefrontal cortex, indicating impaired NVC [232,233]. These effects may stem from dysfunctions in neurons, astrocytes, and endothelial cells. Sleep deprivation lowers neuronal activity and metabolic demand, contributing to reduced CBF and altered vascular tone [234]. Astrocyte dysfunction further disrupts synaptic plasticity and NVC [235]. In OSA, repeated airway obstruction causes intermittent hypoxia and blood pressure surges, compounding the negative impact on cerebral hemodynamics [236,237].

TCD and NIRS studies show that repeated physiological insults in OSA lead to increased variability in CBF and oxygenation, highlighting a volatile hemodynamic environment [238,239]. Chronic intermittent hypoxia contributes to endothelial dysfunction and impaired CVR by disrupting nitric oxide production and promoting oxidative stress [240]. Additionally, autonomic imbalance—marked by heightened sympathetic and reduced parasympathetic activity—further disrupts cerebrovascular regulation in OSA [213].

While sleep deprivation is a known risk factor, prolonged sleep duration has also been linked to increased stroke risk [241]. This may be due to elevated PWV, indicating arterial stiffness, and a higher likelihood of cardiometabolic dysfunction [242]. Additionally, oversleeping is often associated with reduced physical activity [243], contributing to poorer overall health and increased cerebrovascular vulnerability [244].

### 4.2. Changes in Sleep Macrostructure and Microstructure

The relationship between sleep and stroke recovery is complex and multi-dimensional [245]. Emerging evidence highlights the critical role of both sleep macrostructure and microstructure in brain repair and functional recovery. Far from being passive, sleep actively supports memory consolidation, synaptic plasticity, metabolic regulation, and immune modulation [246,247]. Disruptions to these processes after stroke can hinder neural repair and reorganization, ultimately affecting recovery outcomes [245].

Sleep macrostructure refers to the overall architecture of sleep, including wakefulness, NREM stages 1–3, and REM sleep, along with their durations throughout the night [245]. Typically assessed via PSG, it reflects sleep continuity, quality, and stage distribution—key indicators of brain health. Numerous PSG studies have reported significant macrostructural disruptions in stroke patients [248], especially during the acute post-stroke phase [249,250].

A common finding in stroke patients is reduced TST [251], often accompanied by decreased sleep efficiency—the proportion of time asleep while in bed [252]—indicating fragmented and less restorative sleep [249]. These patients also tend to experience increased sleep latency, reflecting difficulty initiating sleep and more frequent arousals, particularly in the acute post-stroke phase [253,254].

Stroke can alter specific sleep stages, particularly SWS, NREM stage 3, and REM [245]. SWS, marked by high-voltage slow brain waves, supports memory consolidation, synaptic plasticity, and metabolic waste clearance [247]. Studies show a consistent reduction in SWS duration and percentage in stroke patients, potentially impairing these restorative functions and slowing recovery [252].

The impact of stroke on REM sleep remains unclear. Some studies report reduced REM sleep in acute stroke patients, suggesting a link to stroke severity [254], while others find no consistent changes [249]. This inconsistency underscores the limitations of sleep macrostructure analysis, which, though informative about stage durations, does not capture the brain’s detailed electrical activity—highlighting the importance of examining sleep microstructure for a deeper understanding [245].

Sleep microstructure refers to the subtle, fine-grained electrical activity of the brain during sleep that may be overlooked by conventional staging. Quantitative EEG is a powerful tool for analyzing microstructure, enabling objective measurement of brain wave frequencies and revealing patterns of activity across sleep stages that may influence key physiological processes [255].

qEEG studies in stroke patients consistently show reduced delta and sigma power during NREM sleep [255]. Lower delta activity reflects impaired deep sleep, potentially affecting restorative functions and synaptic plasticity [252]. Reduced sigma power, indicating fewer sleep spindles [255]—brief 11–16 Hz bursts during stage 2 NREM—suggests deficits in memory consolidation and sensory gating [256]. These impairments may hinder recovery by disrupting core sleep-dependent brain processes [257].

Beyond spindle density, examining spindle characteristics such as amplitude and duration may offer deeper insights into the link between sleep microstructure and stroke recovery [258,259]. Studies have shown that reduced spindle density and amplitude are associated with a higher risk of developing hypertension—a major complication in stroke patients [258]. Studies have shown that reduced spindle density and amplitude are associated with a higher risk of developing hypertension—a major complication in stroke patients.

These findings underscore the critical role of sleep microstructure in stroke recovery and highlight the need for a more comprehensive research approach [245]. While macrostructural and microstructural analyses each provide valuable insights, neither alone captures the full spectrum of neurophysiological changes during sleep. A combined approach is essential for identifying sleep features that predict recovery outcomes and for developing targeted interventions to enhance sleep quality and support brain healing and reorganization in stroke patients [245].

## 5. Screening for Sleep Disorders

Smoking, obesity, and alcohol use are established risk factors for OSA, along with conditions like diabetes, hypertension, coronary artery disease, and prior stroke [8]. OSA is more prevalent in men and older adults [8]. Screening tools such as STOP-BANG, the Berlin Questionnaire, and NoSAS assess risk based on factors like blood pressure, BMI, neck circumference, snoring, and daytime tiredness, though their specificity is limited [260]. High-risk individuals are referred for PSG—the diagnostic gold standard—which records EEG, chin EMG, and EOG to assess sleep stages and arousals. OSA severity is classified using the AHI: 5–14 (mild), 15–29 (moderate), and ≥30 (severe), based on the number of apnea and hypopnea events per hour of sleep. Home sleep testing is also an option for patients with a high pretest probability of OSA [260].

Stroke is a leading cause of long-term disability and death, with a high risk of recurrence, posing a significant public health burden [261]. Given the complex, bidirectional relationship between sleep and stroke, improving our understanding of both may enhance prevention and management strategies [262]. Evidence shows that sleep disorders—particularly OSA—not only commonly affect stroke survivors but also independently increase the risk of stroke onset and recurrence [263,264]. Despite strong recommendations from the American Heart Association/American Stroke Association to conduct sleep studies in all acute stroke and TIA patients, screening rates remain low [265]. This gap is largely due to limited awareness among stroke care providers about the prevalence and impact of sleep disorders [266]. A more proactive approach to screening, especially in high-risk individuals, is urgently needed [267].

The need for comprehensive sleep screening in stroke care extends beyond OSA. While most research has focused on OSA, growing evidence links other sleep disturbances—such as insomnia, abnormal sleep duration, and circadian rhythm disorders—to increased stroke risk and poorer outcomes [268,269]. This highlights the importance of broadening screening efforts to capture the full spectrum of sleep-related factors. An inclusive strategy should assess comorbid sleep disorders like insomnia, RLS, and periodic limb movement disorder using validated tools such as the Insomnia Severity Index [270], the International RLS Study Group Rating Scale [271], and the ESS [272], which can be integrated into routine stroke assessments.

Second, combining multiple screening methods—such as self-report questionnaires, clinical interviews, actigraphy, and portable sleep monitors—can enhance the identification of sleep disorders [273]. Third, tailoring screening strategies to individual patient characteristics, including stroke severity, age, and comorbidities, may further improve detection [274]. For example, patients with brainstem strokes may benefit from targeted assessments for RBD [96].

Advancements in technology, particularly artificial intelligence and machine learning, offer new opportunities for screening sleep disorders [275]. AI can analyze clinical data, vital signs, and electronic health records to identify patients at high risk for OSA and other sleep disturbances. Additionally, targeted education and training for stroke providers on the epidemiology and impact of sleep disorders are essential. Emphasizing comprehensive sleep assessments, with clear screening and referral protocols, can enhance clinicians’ ability to detect and manage sleep-related issues in stroke patients. A multifaceted, technology-driven approach may improve outcomes and reduce the overall burden of stroke.

## 6. Management

Management of symptomatic OSA includes lifestyle modifications, medical devices, pharmacotherapy, and surgery. Behavioral interventions such as weight loss, regular exercise, reduced alcohol intake, and avoiding the supine sleep position can significantly improve OSA symptoms. PAP therapy remains the gold standard, typically involving a nasal or oronasal mask worn during sleep. Surgical interventions targeting the upper airway are considered for patients unresponsive to conservative treatments [276]. Pharmacological options include agents that enhance wakefulness—such as modafinil, pitolisant, and solriamfetol—and those that improve upper airway muscle tone, including atomoxetine, reboxetine, and oxybutynin. Other drug classes under investigation include dopamine and norepinephrine reuptake inhibitors, adrenergic-anticholinergic combinations, and orexin agonists [277].

In iron-deficient patients with RLS, oral iron therapy—often combined with vitamin C to enhance absorption—is a first-line treatment. Recent guidelines now recommend gabapentin extended-release as the preferred initial pharmacologic therapy due to the risk of augmentation associated with long-term use of dopamine agonists such as pramipexole, ropinirole, and rotigotine [278]. Other effective options include alpha-2-delta ligands like gabapentin and pregabalin, which have shown efficacy in clinical trials. In refractory cases, low-potency opioids such as tramadol may be considered under careful supervision. Additionally, medications like atomoxetine and oxybutynin are being explored for their potential to modulate neural pathways involved in RLS symptoms [279].

Clonazepam and immediate-release melatonin remain the first-line treatments for RBD, with melatonin favored for its lower side effect profile. If monotherapy is ineffective, combination therapy or adjunctive use of agents such as carbamazepine or pramipexole may be considered, though evidence for their efficacy is limited. Dopamine agonists like pramipexole, ropinirole, and rotigotine, as well as cholinesterase inhibitors such as rivastigmine, have been explored in specific contexts, particularly in patients with comorbid neurodegenerative conditions. However, selective serotonin reuptake inhibitors (e.g., paroxetine) and levodopa are generally not recommended, as they may exacerbate RBD symptoms [280].

Pharmacologic treatments for insomnia include antihistamines, benzodiazepines, nonbenzodiazepine hypnotics, orexin/hypocretin receptor antagonists, and melatonin receptor agonists. Recent guidelines emphasize the use of dual orexin receptor antagonists for their efficacy and safety profile [281]. For EDS and narcolepsy, FDA-approved therapies include stimulants (e.g., dextroamphetamine, methylphenidate), wake-promoting agents (e.g., modafinil, armodafinil, solriamfetol, pitolisant), and sodium oxybate. Notably, LUMRYZ, an extended-release sodium oxybate, has been approved for pediatric narcolepsy, offering once-nightly dosing. These treatments target both EDS and cataplexy, improving quality of life and adherence (Table S8 [277,279,280,282,283,284,285,286,287,288,289,290,291,292,293,294,295,296,297]).

CPAP is the first-line treatment for OSA in stroke patients [298]. It has been shown to reduce excessive daytime sleepiness, improve quality of life, and lower blood pressure [188]. While recent randomized trials have not consistently demonstrated CPAP’s efficacy in secondary stroke prevention, post hoc analyses suggest that patients who adhere to CPAP therapy for more than 4 h per night may experience reduced cardiovascular events [299]. CPAP also shows promise in enhancing cognitive function and functional recovery after stroke [300]. However, adherence remains a significant challenge in this population. Early initiation of CPAP—within 7 days of stroke—may further improve neurologic outcomes [262].

Management of CSA after stroke depends on the severity and underlying pathophysiology. Nocturnal hypoxemia is commonly addressed with supplemental oxygen therapy [301]. Adaptive servo-ventilation (ASV) is recommended for patients with persistent CSA, particularly when associated with Cheyne–Stokes respiration, and it has shown promising results in post-stroke populations [302]. In severe cases, noninvasive or mechanical ventilation may be necessary to ensure adequate respiratory support. Treatment should be individualized, considering comorbidities, stroke severity, and patient tolerance [303].

Post-stroke insomnia is best managed through a multi-component approach that addresses contributing factors such as depression, anxiety, and fatigue [65]. Cognitive behavioral therapy for insomnia (CBT-I), available in both in-person and digital formats, is the first-line treatment and has been shown to significantly improve sleep quality and reduce insomnia symptoms [81]. Bright light therapy [304], particularly when combined with antidepressants like escitalopram [305], has demonstrated additional benefits in improving sleep and mood. Acupuncture has also shown promise in reducing sleep latency and enhancing sleep efficiency in post-stroke patients [306].

Post-stroke hypersomnia, often presenting as excessive daytime sleepiness (EDS), can be managed through both pharmacological and non-pharmacological approaches. Stimulants such as modafinil have shown potential in improving wakefulness and reducing fatigue, though further research is needed to confirm their efficacy and safety in stroke populations [307]. Non-pharmacological strategies, including good sleep hygiene and regular physical activity, are also effective and widely recommended [308].

The most commonly prescribed treatments for stroke-related RLS include iron supplementation, gabapentin, and dopaminergic agents. According to the 2025 AASM guidelines, iron evaluation is essential for all patients with RLS, with intravenous ferric carboxymaltose strongly recommended for those with low iron stores, and oral ferrous sulfate conditionally recommended [309]. Gabapentin, an alpha-2-delta ligand, is effective for symptom relief and may also benefit patients with concurrent post-stroke neuropathic pain [310]. While dopamine agonists such as pramipexole and ropinirole have historically improved RLS symptoms and sleep quality, their use is now conditionally discouraged due to the risk of augmentation with long-term use [311].

Management of RBD after stroke focuses primarily on injury prevention and patient safety. The 2025 AASM guidelines recommend clonazepam and immediate-release melatonin as first-line treatments, with melatonin favored in older adults due to its safer side effect profile. Clonazepam can reduce dream enactment behaviors but requires caution in elderly or cognitively impaired patients due to sedation risks [96]. Melatonin has shown efficacy in reducing violent behaviors during REM sleep and improving sleep quality. However, further research is needed to confirm the long-term safety and effectiveness of these treatments specifically in post-stroke populations [312]. Environmental modifications, such as removing hazardous objects from the bedroom, are also strongly advised to prevent injury.

After stroke, circadian rhythm disturbances may be addressed through morning bright light therapy, melatonin supplementation, and improved sleep hygiene. Bright light exposure can help realign the circadian pacemaker and enhance sleep–wake regulation [18]. While melatonin has shown promise in improving circadian function and sleep quality in animal stroke models, its efficacy in humans remains insufficiently studied [313].

### CPAP Compliance in Stroke Patients

Long-term adherence to PAP therapy ranges from 65% to 80% after four years [276]. Adherence improves with patient education on the benefits of PAP and risks of obstructive sleep apnea (OSA), as well as behavioral strategies like cognitive behavioral therapy (CBT) and motivational enhancement. The Centers for Medicare & Medicaid Services defines adherence as using PAP for ≥4 h per night on ≥5 nights per week. In a cohort of 2.6 million patients initiating PAP between 2014 and 2017, 75% met this criterion, with an average use of 6 h per night on 93% of nights [276].

Some studies suggest that CPAP therapy may enhance stroke rehabilitation outcomes [314,315]. However, initiating CPAP soon after stroke is challenging, with adherence rates as low as 12–25% [316]. Stroke patients with OSA differ from the general OSA population, often presenting with lower body weight and fewer classic symptoms like excessive sleepiness or snoring [317,318]. They also experience greater functional impairments—such as limb weakness, dysphagia, aphasia, and cognitive deficits—which can reduce CPAP tolerance. Psychological factors like post-stroke depression, anxiety, fatigue, and pain further complicate adherence [262]. The Sleep Apnea Cardiovascular Endpoints (SAVE) trial (NCT00738179), a large multicenter RCT, is evaluating whether CPAP reduces cardiovascular events in patients with CVD and moderate-to-severe OSA. Outcomes are expected to be closely tied to CPAP adherence [319].

Although CPAP effectively eliminates SDB events and alleviates OSA symptoms, long-term adherence remains a challenge. Nonadherence—defined as using CPAP for less than 4 h per night—has been reported in 29% to 83% of patients [320]. Greater nightly usage is associated with improved OSA symptoms, reduced daytime sleepiness, better quality of life, and lower blood pressure [321,322]. Evidence suggests that adherence patterns are often established within the first week of therapy [323,324]. However, no single factor consistently predicts CPAP use, and demographic variables such as age, sex, and marital status have not shown reliable associations with adherence [325]. Instead, adherence appears to be influenced by a complex, individualized set of factors.

Studies show limited and inconsistent correlations between CPAP adherence and OSA severity, as measured by AHI, ODI, or daytime sleepiness [326,327]. However, there is a lack of consistent evidence to support this claim [323]. The SAVE trial, which began in China and later expanded to Australia and New Zealand, highlights the need for more diverse population data. While African American patients have shown lower adherence compared to Caucasians, little is known about CPAP usage patterns in other ethnic groups. One study in Chinese patients found that a higher baseline AHI was the only independent predictor of better CPAP adherence at 1 and 3 months [328].

## 7. Future Studies

Sleep disorders both increase the risk of stroke and are common consequences of stroke, yet they remain underdiagnosed and undertreated in post-stroke populations. Improving diagnostic tools—such as expanding access to polysomnography (PSG)—is essential, though PSG remains costly and limited in availability. Many studies exploring the sleep–stroke relationship are constrained by small sample sizes and observational designs. Raising clinician awareness and enhancing patient education are critical to improving diagnosis and treatment. As research advances, the complex bidirectional relationship between sleep disorders and stroke continues to emerge as a key area requiring nuanced, multidisciplinary approaches.

### 7.1. Beyond AHI: Unmasking Hidden Stroke Predictors

The AHI has long been the standard for diagnosing OSA and assessing cardiovascular risk. However, recent studies challenge its sufficiency, revealing that AHI alone may not capture the full spectrum of sleep-related stroke risk. The DREAM cohort identified seven independent polysomnographic phenotypes associated with cardiovascular outcomes, including stroke, highlighting the need for broader metrics such as hypoxic burden, ODI, and HRV in risk stratification [329].

Emerging evidence also suggests that reduced HRV may serve as a sensitive marker for stroke risk in OSA patients. Additionally, quantitative EEG studies have identified specific brain wave alterations—such as changes in delta, alpha, and beta power, and ratios like DAR and DTABR—that may indicate increased vulnerability to ischemia, even in individuals without diagnosed OSA [72].

### 7.2. Personalized Sleep Phenotypes: Tailoring Interventions to Individual Needs

Sleep disorders are increasingly recognized as heterogeneous conditions with distinct phenotypes and variable stroke risk profiles. This understanding supports a shift toward personalized medicine in stroke care. For example, Ye et al. identified three OSA phenotypes based on symptom clusters, with the “minimal symptom” group paradoxically showing higher odds of hypertension and cardiovascular disease [330].

Similar findings from international studies, including SAGIC, confirm the existence of multiple OSA subtypes across populations, reinforcing the need for individualized treatment strategies based on sleep phenotype [331,332].

### 7.3. Genetics, Race/Ethnicity, and Sex: Unveiling Underlying Vulnerabilities

Genetic research is uncovering loci associated with susceptibility to both sleep disorders and stroke, offering new avenues for risk assessment and targeted interventions. Additionally, race, ethnicity, and sex significantly influence the sleep–stroke relationship. For instance, African American patients have shown lower CPAP adherence, and the study by Appelros et al. highlights how stroke risk and presentation vary by demographic factors, including sex and age. These findings underscore the importance of culturally and biologically tailored public health strategies [333].

### 7.4. Optimizing Treatment: Empowering Patients and Expanding Options

While CPAP remains the gold standard for OSA treatment, adherence challenges have spurred interest in enhancing its effectiveness and exploring alternatives. Behavioral interventions, telemedicine, and smart CPAP technologies have shown promise in improving compliance. For example, Bakker et al. demonstrated that increased motivation correlates with longer CPAP use [334], and Kotzian et al. showed that telemonitoring improves adherence in post-stroke patients [210].

Alternative therapies—such as hypoglossal nerve stimulation, positional therapy, oral appliances, and expiratory positive airway pressure (EPAP)—are being explored for CPAP-intolerant individuals, though more clinical trials are needed to confirm their long-term efficacy and safety in stroke populations.

The timing of OSA treatment post-stroke is also under investigation. Trials like Sleep SMART are evaluating whether early intervention can reduce recurrent stroke risk and improve functional outcomes, potentially reshaping post-stroke care.

### 7.5. Deciphering the Sleep–Stroke Dialogue: Illuminating Mechanisms

Advancements in neuroimaging and electrophysiology are shedding light on how sleep disorders influence stroke risk and recovery. Disrupted sleep has been linked to impaired neurovascular coupling (NVC), altered brain plasticity, and heightened inflammatory responses. Functional imaging studies, such as those by Jensen et al., suggest that CPAP therapy may help restore cerebral blood flow (CBF) in response to hypoxia, potentially normalizing NVC in OSA patients.

Further research using diffusion tensor imaging (DTI) and functional MRI (fMRI) could clarify how OSA treatment influences brain plasticity and recovery post-stroke. In parallel, inflammation is emerging as a key mediator in the sleep–stroke relationship. Monsour et al. highlighted the role of oxidative stress and inflammation in linking OSA to ischemic stroke, suggesting that anti-inflammatory effects of OSA treatment may offer neuroprotective benefits [335].

An exciting frontier is the gut–brain axis. Emerging evidence suggests that the gut microbiome may influence sleep quality, circadian rhythms, and stroke risk. Understanding this relationship could lead to novel, non-invasive interventions targeting microbiota to improve sleep and reduce stroke vulnerability.

## 8. Conclusions

This narrative review highlights the intricate, bidirectional relationship between sleep disorders and stroke. Among these, obstructive sleep apnea (OSA) stands out as a major modifiable risk factor for both stroke onset and recurrence. Pathophysiological mechanisms—such as intermittent hypoxia, inflammation, endothelial dysfunction, and autonomic dysregulation—contribute to increased cerebrovascular vulnerability. Conversely, stroke can disrupt sleep architecture and trigger or exacerbate a range of sleep disorders, including OSA, insomnia, hypersomnia, restless legs syndrome (RLS), and circadian rhythm disturbances.

Despite growing awareness, sleep disorders remain underdiagnosed and undertreated in stroke populations. Significant knowledge gaps persist, particularly regarding non-OSA sleep disorders. Future research must prioritize robust study designs, larger cohorts, and standardized methodologies to clarify causal relationships and quantify risk. Expanding access to diagnostic tools and integrating sleep assessments into routine stroke care are essential steps forward.

Equally important is the education of clinicians and patients about the prevalence and impact of sleep disorders in stroke. Incorporating sleep screening and treatment into post-stroke care guidelines will support early intervention and improve outcomes. Ultimately, a deeper understanding of the sleep–stroke interplay will enable targeted, personalized interventions—empowering patients to reclaim restorative sleep and reducing the global burden of stroke.

## Data Availability

All the data are presented in the manuscript.

## Supplementary Materials

The following supporting information can be downloaded at: https://www.mdpi.com/article/10.3390/medsci13030113/s1, Section S1: Methodology; Figure S1: The diagram illustrates the significant influence that post-stroke sleep disorders have on the recovery process of stroke patients; Figure S2: Pathophysiology of sleep disorders and stroke; Table S1. Prevalence of sleep disorders and their associated stroke risk [5,6,7,8,9,10,11,12,13,14,15]; Table S2: The relationship between OSA and stroke [5,26,27,28,29,30,31,32,33,34]; Table S3: The relationship between insomnia and stroke [68,69,70,71,72,73,74,75,76]; Table S4: The relationship between restless legs syndrome and stroke [86,87,88]; Table S5: Sleep disorders incidence and post-stroke risk [1,38,64,125,126,127,128,129,130,131,132,133,134]; Table S6: Sleep-related breathing disorders after stroke [137,138,139,140,141,142]; Table S7: Sleep-related movement disorders after stroke [124,168,169,170,171,172,173,174,175]; Table S8: Management of sleep disorders associated with stroke [277,279,280,282,283,284,285,286,287,288,289,290,291,292,293,294,295,296,297].

## Abbreviations

The following abbreviations are used in this manuscript:

ABP	Ambulatory blood pressure
ADHD	Attention deficit hyperactivity disorder
AHI	Apnea-hypopnea index
AIS	Acute ischemic stroke
AUC	Area under the curve
BP	Blood pressure
CANTAB	Cambridge neuropsychological test automated battery
CBF	Cerebral blood flow
CCK-8	Cell counting kit-8
CDC	Center for Disease Control and Prevention
CI	Confidence interval
CMC	Chronic medical condition
CPAP	Continuous positive airway pressure
CSA	Central sleep apnea
CSB	Cheyne–stokes breathing
CVD	Cardiovascular disease
CVR	Cerebrovascular reactivity
DAR	Delta/alpha ratio
dCBT	Digital cognitive behavioral therapy
DMN	Default-mode network
DVA	Dynamic vessel analysis
EDS	Excessive daytime sleepiness
EEG	Electroencephalography
EPAP	Expiratory positive airway pressure
ESS	Epworth sleepiness scale
EVT	Endovascular therapy
fMRI	Functional magnetic resonance imaging
fNIRS	Functional near-infrared spectroscopy
GABA	Gamma aminobutyric acid
GLN	Glutamine
GLU	Glutamate
HBPM	Home blood pressure monitoring
HPA	Hypothalamic–pituitary–adrenal
HR	Hazard ratio
IV-tPA	Intravenous tissue plasminogen activator
iRBD	Isolated RBD
MTNR1B	Melatonin receptor type 1B
NIHSS	National institute of health stroke scale
NIRS	Near-infrared spectroscopy
NREM	Non-rapid eye movement
NSD	Nighttime sleep disturbances
NVC	Neurovascular coupling
OSA	Obstructive sleep apnea
PLEDs	Periodic lateralized epileptiform discharges
PLMS	Periodic limb movements of sleep
PSD	Post-stroke depression
PSG	Polysomnography
PSI	Post-stroke insomnia
PSSD	Post-stroke sleep disorder
PWV	Pulse wave velocity
RBD	REM behavior disorder
REM	Rapid eye movement
RLS	Restless legs syndrome
ROS	Reactive oxygen species
SAGIC	Sleep apnea global interdisciplinary consortium
SBP	Systolic blood pressure
SCN	Suprachiasmatic nucleus
SDB	Sleep-disordered breathing
SHHS	Sleep heart health study
SLC6A4	Solute carrier family 6 member 4
SP	Substance P
STOP-BANG	Score for obstructive sleep apnea only
SWS	Slow wave sleep
TCD	Transcranial Doppler
TIA	Transient ischemic attack
TST	Total sleep time
VLPO	Ventrolateral preoptic nucleus
