# Supplementary material for: Sleep Disorders and Stroke: Pathophysiological Links, Clinical Implications, and Management Strategies
Source: Med Sci (Basel). 2025 Aug 5;13(3):113. doi: 10.3390/medsci13030113 (PMC12372127; doi:10.3390/medsci13030113)
Supplement: Supplementary file 1 [file medsci-13-00113-s001.zip › medsci-3708238-supplementary.pdf]

**Supplementary Material**

## Materials

**Sup Mat 1**. Methodology

## Figures

**Figure S1**. The diagram illustrates the significant influence that post-stroke sleep disorders have on the recovery process of stroke patients.

**Figure S2**. Pathophysiology of sleep disorders and stroke.

## Tables

**Table S1**. Prevalence of sleep disorders and their associated stroke risk.

**Table S2**. The relationship between OSA and stroke.

**Table S3**. The relationship between insomnia and stroke.

**Table S4**. The relationship between restless legs syndrome and stroke.

**Table S5**. Sleep disorders incidence and post-stroke risk.

**Table S6**. Sleep-related breathing disorders after stroke.

**Table S7**. Sleep-related movement disorders after stroke.

**Table S8**. Management of sleep disorders associated with stroke.

**Sup Mat 1**

## Methodology

This review was conducted as a narrative review, aiming to synthesize and contextualize the current understanding of the bidirectional relationship between sleep disorders and stroke. The narrative format was chosen to allow for a broad and integrative discussion of diverse sleep disorders, their pathophysiological mechanisms, and their clinical implications in stroke patients.

To ensure a comprehensive and balanced overview, we followed a structured approach to literature selection:

<u>Search Strategy</u>

We conducted a literature search using databases including PubMed, Scopus, and Google Scholar. The search terms included combinations of keywords such as "stroke", "sleep disorders", "obstructive sleep apnea", "insomnia", "restless legs syndrome", "REM sleep behavior disorder", "post-stroke sleep", and "cerebrovascular disease". Searches were limited to articles published in English from 2000 to 2024.

<u>Inclusion And Exclusion Criteria</u>

We included peer-reviewed original research articles, meta-analyses, and systematic reviews involving adult human populations that examined the relationship between sleep disorders and stroke, either as a risk factor or as a consequence, and that provided clinical, epidemiological, or mechanistic insights. We excluded case reports, editorials, non-peer-reviewed sources, and studies focusing exclusively on pediatric populations or animal models unless they were directly relevant to human pathophysiology. Also, studies focusing exclusively on pediatric populations or animal models unless directly relevant to human pathophysiology were excluded.

<u>Selection Process</u>

Titles and abstracts were screened for relevance, followed by full-text review. Reference lists of key articles were also examined to identify additional relevant studies. The final selection was based on relevance, quality, and contribution to the thematic structure of the review.

Data Presentation

Although this is a narrative review, we included summary tables to enhance clarity and accessibility. These tables are not intended to represent a systematic synthesis but rather to organize and highlight key findings from the literature.

Limitations

We acknowledge the inherent limitations of a narrative review, including the potential for selection bias. However, we aimed to mitigate this by using a transparent and reproducible search strategy and by including a diverse range of high-quality sources.

## Figure S1

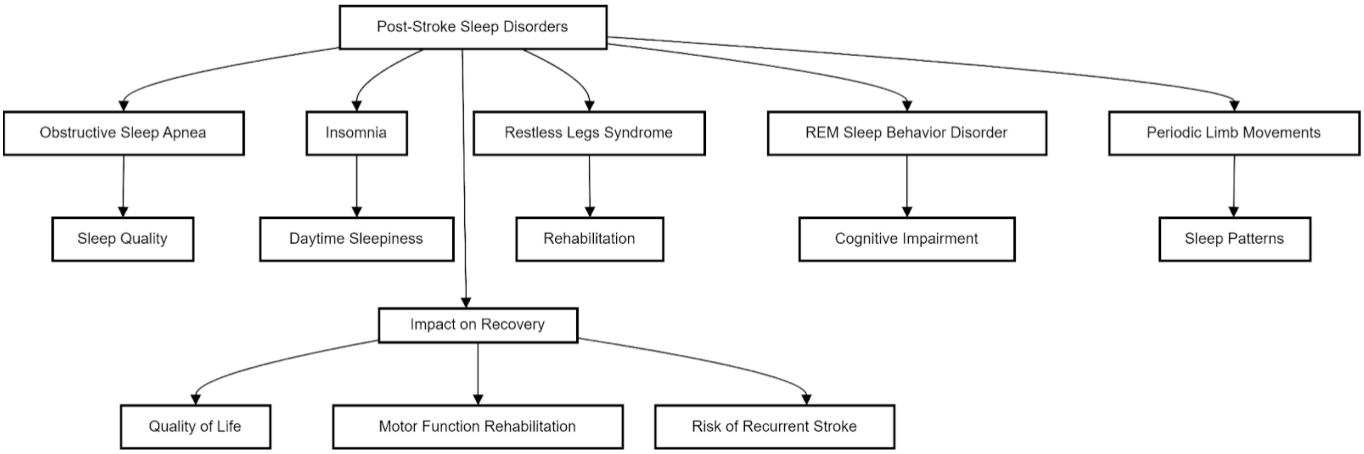


**Figure S1.** The diagram illustrates the significant influence that post-stroke sleep disorders have on the recovery process of stroke patients. It categorizes various sleep disorders, including obstructive sleep apnea (OSA), insomnia, restless legs syndrome (RLS), REM sleep behavior disorder, and periodic limb movements, as critical factors that can impede stroke recovery.

## Figure S2

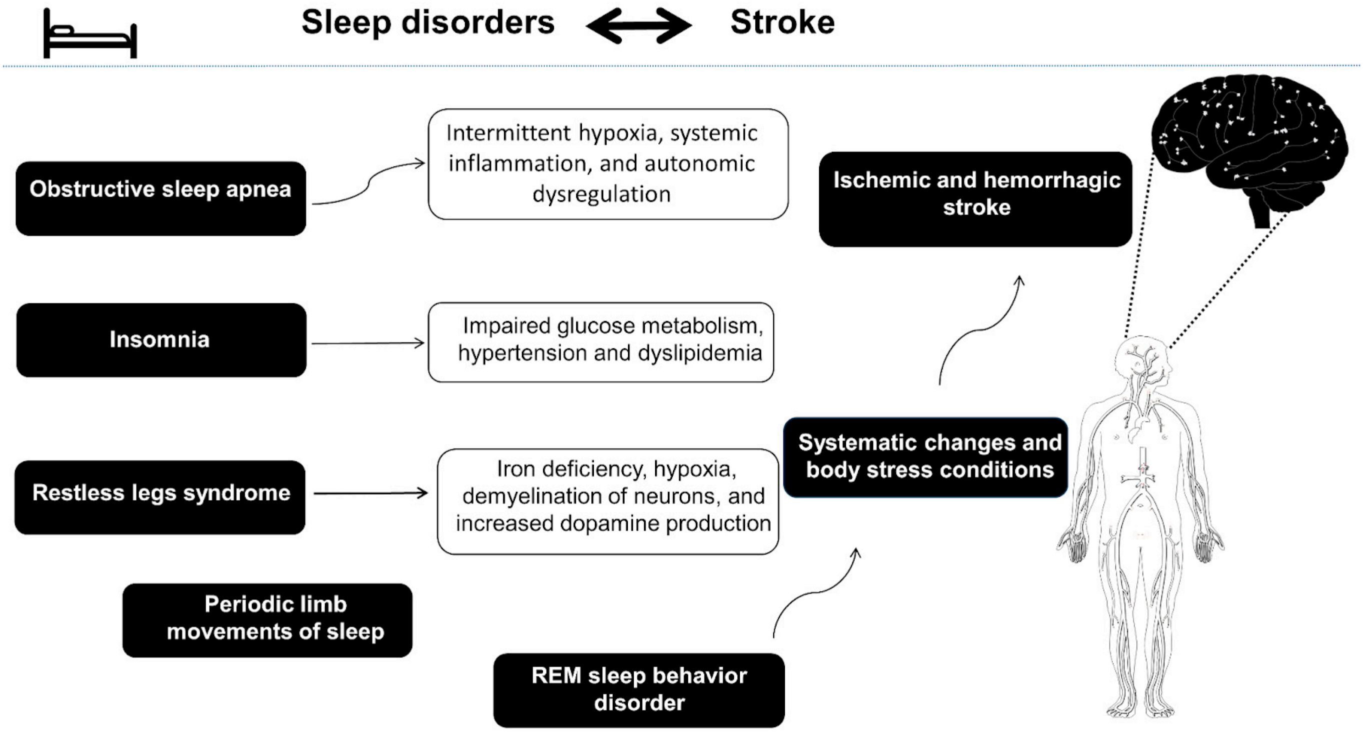


**Figure S2.** Pathophysiology of sleep disorders and stroke. The pathophysiological processes that connect sleep disorders with cerebrovascular events, with a particular focus in this figure on the contributions of intermittent hypoxia, systemic inflammation, autonomic dysregulation in OSA, impaired glucose metabolism, hypertension, dyslipidemia in insomnia and iron deficiency, hypoxia, demyelination of neurons, increased dopamine production in RLS, will lead to systematic alterations and body stress conditions that will finally cause the stroke. At the same time, stroke changes sleeping frequency and breathing patterns, which will cause sleeping disorders and aggravate them, including OSA, insomnia, and RLS.

**Table S1.** Prevalence of sleep disorders and their associated stroke risk.

| Sleep disorder | Prevalence | Risk related to stroke | References |
|---|---|---|---|
| Obstructive sleep apnea | 6% of men (range, 3–18%) and 4% of women (range, 1–17%) | aHR: 2.52, 95% CI: 1.04–6.01 | Munoz et al. (2006) [1]<br>Franklin et al. (2015) [2]<br>05-Aug-25 15:26:00 |
| Insomnia | Variable, around 6.0% in the United States | Pooled HR: 1.15, 95% CI: 1.07–1.24 | Morin et al. (2022) [3]<br>Leng et al. (2015) [4] |
| Restless legs syndrome | Varies, 1–3% reported in Asian countries and 5-13% in Europe and North America | Pooled RR: 1.45, 95% CI: 1.24–1.69 | Liu et al. (2018) [5]<br>Manconi et al. (2021) [6] |
| Rapid eye movement sleep behavior disorder | 3–10% worldwide | aHR: 1.93, 95% CI: 2.27–19.27 | Ma et al. (2017) [7]<br>Dauvilliers et al. (2018) [8] |
| Periodic limb movements of sleep | 4–11% of adults | OR: 6.33<br>RR: 3.8 | Hornyak et al. (2006) [9]<br>Plomaritis et al. (2023) [10]<br>Coelho et al. (2010) [11] |

Abbreviations: aHR, Adjusted Hazard Ratio; CI, Confidence Interval; OR, Odds Ratio; RR, Risk Ratio.

## Table S1

## Table S2

**Table S2.** The relationship between OSA and stroke.

| Reference | Population | Study Design | Key Findings | Risk related to stroke |
|---|---|---|---|---|
| Mohsenin et al. (1995) [12] | 10 stroke patients, 10 controls | Case–Control Study | Stroke patients had abnormal sleep architecture and increased respiratory disturbances. | 80% of the stroke individuals with OSA |
| Arzt et al. (2005) [13] | Wisconsin Sleep Cohort Study* | Prospective Cohort Study | Increased risk of stroke in individuals with moderate to severe OSA. | Moderate OSA: HR 3.0 |
| Munoz et al. (2006) [1] | 394 elderly (70-100 years) | Prospective Longitudinal Study | Severe OSA independently increases the risk of ischemic stroke in the elderly. | Severe OSA: HR 2.52 (95% CI 1.04–6.01) |
| Redline et al. (2010) [14] | 5,422 participants without a history of stroke | Longitudinal Cohort Study | There is an association between stroke and OAHI in men but not in women. Increased risk of stroke in severe OSA (AHI ≥ 30) compared to no OSA (AHI < 5). | aHR: 2.24 (95% CI: 1.05–4.78). |
| Young et al. (2010) [15] | the cohort expanded to include 1550 men and women. | Longitudinal Study | Severe OSA significantly increases the risk of incident stroke. Increased odds of stroke in severe OSA (AHI ≥ 30) compared to no OSA (AHI < 5). | aOR: 2.94 (95% CI: 1.07–8.11). |
| Wang et al. (2013) [16] | Meta-analysis of 12 studies (25,760 participants) | Meta-Analysis | Severe OSA significantly increases the risk of stroke and all-cause mortality. | RR with an AHI of <5 was 2.15 (95% CI: 1.42–3.24) for incident fatal and non-fatal stroke. |
| Hsieh et al. (2019) [17] | LHID study** | Population-Based Cohort Study | Increased risk of stroke in moderate to severe OSA compared to no OSA. | aHR: 1.42 (95% CI: 1.19–1.69). |
| Haula et al. (2021) [18] | 95 TIA and mild to moderate stroke patients | Observational Prospective Longitudinal Study | Patients with wake-up stroke (WUS) are more likely to have moderate-to-severe OSA compared to non-WUS patients. | OR: 2.61; RR: 1.68 |

| | | | | |
|---|---|---|---|---|
| Edrissi et al. (2022) [19] | 5,469 AIS patients (2,662 men, 2,807 women) | Retrospective Cohort | Gender differences in AIS patients with OSA, with women presenting more severe comorbidities. A total of 170 AIS patients presented with a baseline OSA. 101 were men (59.41%), and 69 were women (40.59%). | Women are more likely to present with PVD: OR = 4.892 (95%, CI: 1.248–19.2, *p*=0.023). Depression: OR = 3.804 (95%, CI = 1.369–10.6, *p*=0.01). Higher BMI: OR = 1.103 (95%, CI: 1.036–1.18, *p*=0.002). Higher HDL levels: OR = 1.125 (95%, CI: 1.067–1.19, *p*<0.001). No significant clinical risk factors were associated with men with AIS and OSA. |
| Robbins et al. (2024) [20] | 22,192 participants (38.1% Black) | Population-Based Cohort Study | White individuals with high OSA risk and PDSA were at increased incident stroke risk. PAP therapy use was associated with reduced stroke risk in Black individuals with PDSA. | White: High OSA risk HR 1.22 (95%, CI: 1.01–1.47); PDSA HR 1.33 (95%, CI: 1.04–1.70); PAP therapy HR 1.38 (95%, CI: 1.05–1.80); Black: PDSA + PAP therapy HR 0.39 (95%, CI: 0.17–0.91) |

**Abbreviations**: AIS: Androgen Insensitivity Syndrome; AHI: Apnea–Hypopnea Index, a measure of OSA severity; BMI: Body Mass Index; CI: Confidence Interval; CVD: Cardiovascular Disease; HDL: High-Density Lipoprotein; HR: Hazard Ratio; OAHI: Obstructive Apnea–Hypopnea Index; OR: Odds Ratio; OSA: Obstructive Sleep Apnea; PAP: Positive Airway Pressure; PDSA: Provider-Diagnosed Sleep Apnea; PVD: Peripheral Vascular Disease; RR: Risk Ratio; TIA: Transient Ischemic Attack; WUS: Wake-Up Stroke.

*Wisconsin Sleep Cohort Study: The study population consists of Wisconsin state employees aged 30-60 years in 1988. The cohort included both high-risk respondents (those reporting snoring sometimes or more frequently, or very loud snoring, or witnessed breathing pauses) and a random sample of low-risk respondents.

**Longitudinal Health Insurance Databases (LHID2000, LHID2005, LHID2010): These datasets include a random sample of 1 million beneficiaries from the original NHIRD for the years 2000, 2005, and 2010, respectively.

## Table S3

**Table S3.** The relationship between insomnia and stroke.

| Reference | Population | Study Design | Key Findings | Risk related to stroke (OR, RR) |
|---|---|---|---|---|
| Elwood et al. (2006) [21] | 1,986 men aged 55-69 years | Prospective cohort study | Up to one-third of the men reported at least one symptom of sleep disturbance, and one-third reported daytime sleepiness. | The risk of ischemic stroke was increased in men with any sleep disturbance, with the strongest association observed in men with OSA OR 1.97 (95%, CI: 1.26–3.09). |
| Jaussent et al. (2013) [22] | 2773 participants | Prospective cohort study | Insomnia symptoms associated with increased risk of incident stroke. | OR 1.37 (95%, CI: 1.12–1.67) |
| Liu et al. (2022) [23] | 31,126 participants | Bidirectional meta-analysis of prospective cohort studies | Insomnia is associated with an increased risk of hypertension, which is a risk factor for stroke. | aOR 1.20 (95%, CI: 1.08–1.32) |
| Sands-Lincoln et al. (2013) [24] | 71,617 middle-aged women | Prospective cohort study | Persistent insomnia symptoms are associated with a higher risk of stroke mortality | Adjusted HR for stroke mortality: 1.38 (95% CI: 1.09–1.75) |
| Wu et al. (2014) [25] | 85,752 adults without a previous diagnosis of stroke or OSA | Retrospective cohort study | The risk of stroke was most pronounced among younger adults aged 18 to 34 years, with an incidence rate ratio of 8.06 compared to their non insomniac counterparts. | Patients with insomnia had a 54% higher risk of developing stroke compared to non-insomnia patients with aHR 1.54 (95%, CI: 1.38–1.72). |
| Hsu et al. (2015) [26] | 44,080 individuals, adults, without a previous diagnosis of stroke, coronary heart disease, or OSA | Population-based study | Insomnia is associated with a higher prevalence of stroke and TIA. | HR 1.85; ($p$<0.001). |
| Zheng et al. (2019) [27] | 487,200 adults without a previous diagnosis of stroke, coronary heart disease, or cancer. | Prospective cohort study | Risk of total stroke, hemorrhagic stroke, and ischemic stroke. Associations of any insomnia symptoms and number of insomnia symptoms with risk of stroke. | All three symptoms (DIMS, EMA, and DDF) were associated with slightly increased risks of total stroke incidence HR 1.05 (95%, CI: 1.05–1.08) and ischemic stroke incidence HR 1.06 (95%, CI: 1.07–1.09). No associations were observed between the 3 symptoms and |

| | | | | |
|---|---|---|---|---|
| | | | | hemorrhagic stroke incidence. Compared with those with no insomnia symptoms, 1, 2, or 3 symptoms increases the risk of stroke by 7, 10, and 18%, respectively. |
| Xu et al. (2023) [28] | 508 patients diagnosed with AIS who underwent EVT for large vessel occlusion | Single-center, observational, case–control study | The incidence of insomnia in the study population was 39.6% (n = 144, insomnia group; n = 364, non-insomnia group). The insomnia group exhibited higher levels of serum inflammatory markers (CRP, white blood cell count, neutrophil count) and oxidative stress biomarkers (HIF-1$\alpha$, MDA). | Compared with the non-insomnia group, a worse prognosis outcome was observed in 63% vs. 49% with aRR 1.8 (95%, CI: 1.2–3.7; $p$=0.016) |
| Geusgens et al. (2024) [29] | 66 outpatient stroke survivors who were three to six months post-stroke | Cross-sectional observational study | Insomnia and pre-stroke depression treatment were significant predictors of post-stroke depression. A significant positive correlation was found between insomnia severity and depression scores. | Participants with more insomnia complaints had higher depression scores post-stroke ($\beta = 0.48$, $t = 4.40$, $p < 0.001$). Participants who had received pre-stroke depression treatment also had higher depression scores post-stroke ($\beta$=0.24, t=2.28, $p$=0.026). |

**Abbreviations:** CI: Confidence Interval; CRP: C-Reactive Protein; DDF: Daytime Dysfunction; DIMS: Difficulty in Initiating or Maintaining Sleep; EMA: Early Morning Awakening; EVT: Endovascular Treatment; HIF-1$\alpha$: Hypoxia Index Factor 1$\alpha$; HR: Hazard Ratio; ICD-9-CM: International Classification of Diseases, Ninth Revision, Clinical Modification; IRR: Incidence Rate Ratio; IS: Ischemic Stroke; KoGES: Korean Genome and Epidemiology Study; MDA: Malondialdehyde; OR: Odds Ratio; TIA: Transient Ischemic Attack; UK: United Kingdom.

# Table S4

**Table S4.** The relationship between restless legs syndrome and stroke.

| Reference | Population | Study Design | Key Findings | Risk related to stroke (OR, RR) |
|---|---|---|---|---|
| Janes et al. (2021) [30] | 44 patients with RLS | Case–control study | Mean IMT was significantly increased in patients with RLS when measured immediately proximally to carotid bifurcation versus controls. Patients showed higher CBFVs compared to controls. After multivariate analysis, age, hypertension, and RLS proved to be independent IMT predictors. | NA |
| Kalampokini et al. (2022) [31] | 19 patients with RLS | Systematic review and meta-analysis | The symptoms occurred simultaneously with the stroke or a few days after. The most common location of stroke was the pons and less commonly the medulla. In most cases, symptoms were unilateral. In the majority of those cases, the contralateral limb was affected due to a lateral pons infarction. RLS symptoms after infarction improved or resolved in most cases within a few days to 3 months. In almost all patients who received dopaminergic treatment, the symptoms improved significantly or resolved completely. | In 11 out of 19 cases, one limb was affected, which in most of those cases (10 out of 11, 91%) was the contralateral due to a lateral brainstem lesion. |
| Schlesinger et al. (2015) [32] | 22 of 149 patients (15%) and 10 of 298 controls (3%) suffered from RLS | Case–control study | Patients hospitalized with acute stroke/TIA for the presence of RLS. There was a significant association between RLS and stroke/TIA. This association remained after adjustment for common cardiovascular risk factors. | RLS among patients with stroke/TIA versus controls was OR 7.60 (95%, CI: 2.07–27.87 |

Abbreviation: CBFVs: Cerebrovascular Flow Velocities; CI: Confidence Interval; IMT: Intima-Media Thickness; OR: Odds Ratio; RR: Risk Ratio.

## Table S5

**Table S5.** Sleep disorder incidence and post-stroke risk.

| Sleep disorder | Incidence | Post-stroke risks | Reference |
|---|---|---|---|
| OSA | In middle-aged adults with AHI ≥ 5, OSA was 9% for women and 24% for men | 71% in subjects with AHI greater than 5/h. | Young et al. (1993) [33]<br>Seiler et al. (2019) [34]<br>Hermann et al. (2016) [35] |
| Circadian rhythm disorders | As many as 92.4% of patients with mild to moderate ischemic strokes suffer from acute sleep disorders | Patients with disrupted circadian rhythms post-stroke have a higher risk of poor outcomes, including higher mortality rates and increased disability. | Gan et al. (2018) [36]<br>Kamat et al. (2023) [37] |
| Insomnia | 30% in population-based survey, and 10% according to NIH diagnostic criteria | 18% new-onset insomnia. | Ancoli-Israel et al. (1999) [38]<br>NIH et al. (2005) [39]<br>Hepburn et al. (2018) [40] |
| Hypersomnia | 20-40% of stroke patients | Hypersomnia is commonly observed in stroke survivors and has been linked to poorer outcomes, including increased mortality and disability rates. | Vock et al. 2002 [41]<br>Jang et al. 2016 [42] |
| Sleep-related movement disorders (RLS) | Occurring in 10–15% of stroke survivors | It was found that 29.4% of post-stroke patients with RLS had no involvement of the limbs during the acute phase of the stroke. Another 29.4% had contralateral RLS, whereas the rest had bilateral symptoms. | Hermann et al. (2009) [43]<br>Sechi et al. (2008) [44] |
| Depression | Nearly 30% of stroke patients develop depression | Depression can further impede recovery by reducing motivation and increasing feelings of hopelessness. | Paolucci et al. (2008) [45] |

Abbreviations: AHI: Apnea–Hypopnea Index; OSA: Obstructive Sleep Apnea; RLS: Restless Legs Syndrome.

## Table S6

**Table S6.** Sleep-related breathing disorders after stroke.

| Reference | Population | Study Design | Key Findings | Risk related to stroke (OR, RR) |
|---|---|---|---|---|
| Boulos et al. (2016) [46] | 69 patients with stroke or TIA | Observational study | Thirty-two patients (46.4%) were found to have OSA. Male sex, BMI, and atrial fibrillation were independent predictors of OSA. | Stroke/TIA patients are 2.32 times more likely to have OSA compared to the general population. Also, stroke/TIA patients are about 3.40 times more likely to have OSA compared to the general population. |
| Dharmakulaseelan et al. (2023) [47] | 171 patients | Retrospective evaluation of data from three previously conducted studies | Female sex was an independent predictor of lower OSA severity as measured by AHI ($\beta = -5.93$, 95% CI −11.21– −0.66). | Females with post-stroke OSA had poorer functional outcomes and more severe strokes compared to males despite having lower OSA severity. |
| Klingman et al. (2024) [48] | 123 participants who were approximately 15 days post-stroke | Cross-sectional descriptive study | The prevalence of undiagnosed OSA in the early stroke recovery phase was 22%, and AUC was 0.746 ($p$<0.001), indicating the BASH tool is an effective screener for OSA in this population. | Participants with OSA have RR 2.67 and OR 4.75 times of experience stroke. |
| Rangel et al. (2024) [49] | 65 individuals with chronic stage stroke (at least 6 months since the stroke onset) | Prospective exploratory study | 52.4% of the stroke participants reported insomnia symptoms. 33.9% reported EDS, which is often related to poor sleep quality and insomnia. | Stroke participants are approximately 4.39 times more likely to report insomnia symptoms compared to non-stroke individuals. Stroke participants are approximately 2.26 times more likely to have poor sleep quality compared to non-stroke individuals. |
| Yang et al. (2024) [50] | 2837 stroke patients | Systematic review | The incidence of OSA in stroke patients increased from 61% in 2011 to 75% in 2019. Compared to the general population, the incidence of OSA is significantly higher in stroke patients (75%) compared to the general population (35%). | Patients with stroke were 5.58 times more likely to have OSA compared to non-stroke individuals. |

| | | | | |
|---|---|---|---|---|
| Yang et al. (2024) [51] | 227 eligible patients with OSA post-stroke. | Prospective observational study | Short-term CPAP adherence rate was positively associated with being married or living with a partner; higher educational attainment (associate degree or higher); stronger health beliefs; long-term CPAP adherence rate among those adherent short-term: 25% (n = 19) | Married or living with a partner are approximately 2.69 times more likely to adhere to CPAP than those who are single or widowed. Participants with an associate degree or higher are approximately 2.24 times more likely to adhere to CPAP than those with a lower education level. |

Abbreviations: BASH B: Body Mass index; A: Age; S: Sex; H: Hypertension; BMI: Body Mass Index; EDS: Excessive daytime sleep; CPAP: Continuous Positive Airway Pressure; OSA: Obstructive Sleep Apnea; TIA: Transient Ischemic Attack; CI: Confidence Interval; OR: odds ratio; RR: risk ratio; AUC: area under the curve.

# Table S7

**Table S7.** Sleep-related movement disorders after stroke.

| **Reference** | **Population** | **Study Design** | **Key Findings** | **Risk related to stroke (OR, RR)** |
|---|---|---|---|---|
| Boulos et al. (2017) [52] | 23 patients with RLS | Prospective cohort study | Patients sustaining minor stroke or high-risk TIA, a concurrent diagnosis of RLS was independently associated with worse QoL both at baseline and 2–6-month follow-up. | OR (at baseline) 0.28 (95%, CI: 0.10–0.74). OR (after 6 months of follow-up) 0.14 (95%, CI: 0.02–0.82). |
| Gupta et al. (2017) [53] | 35 patients with RLS | A prospective study | RLS, especially unilateral or asymmetrical, might frequently pre-exist in patients presenting with subcortical stroke. | Of 346 stroke patients, 35 (10.11%) were diagnosed with RLS. |
| Woo et al. (2017) [54] | 6 patients with psRLS or psPLMS | Retrospective study | Post-stroke patients exhibit a higher prevalence of RLS and PLMS compared to the general population.<br>In post-stroke patients, PLMS can often be unilateral, affecting only one side of the body. Both RLS and PLMS significantly disrupt sleep, leading to poorer sleep quality and increased daytime sleepiness in post-stroke patients. | NA. |
| Shiina et al. (2019) [55] | 8 patients had RLS | Prospective study | Stroke can trigger the onset or exacerbate existing symptoms of RLS. RLS symptoms can interfere with the rehabilitation and recovery process post-stroke. | Among 104 patients with AIS, 6 (5.8%) and 2 patients (1.9%) had RLS and RLS variants, respectively. Three (3.3%) |

| | | | | |
|---|---|---|---|---|
| | | | | had post-stroke RLS/RLS variants: 2 (66.7%) had bilateral symptoms, and 1 (33.3%) had unilateral symptoms contralateral to the lesion. |
| Han et al. (2019) [56] | 16 patients with RLS | Observational study | RLS has been associated with worse clinical outcomes in stroke patients. This includes more severe initial stroke presentations and poorer functional outcomes during recovery. The presence of RLS in stroke patients may contribute to an increased risk of subsequent strokes and other cardiovascular events. | Of 296 patients with AIS, 16 (5.4%) were diagnosed with RLS. Diagnosis of RLS in patients with AIS was associated with poor clinical outcome three months after stroke (mRS 3-6) (OR 4.26; 95%, CI: 1.22–14.79, *p*=0.022) along with initial NIHSS score. |
| Zhang et al. (2019) [57] | 22 patients with RLS | Prospective study | A higher prevalence of RLS is observed in ischemic stroke patients compared to the general population. The presence of RLS may correlate with poorer functional outcomes and slower recovery post-stroke. Stroke patients with RLS might have a higher risk of recurrent strokes and cardiovascular events. | Subcortical infarction was identified as a risk factor for RLS in ischemic stroke patients (OR 4.05; 95%, CI: 1.15–14.18, *p*<0.05). 22 cases of RLS were identified among 199 ischemic stroke patients (11.1%). |
| Hasan et al. (2021) [58] | 15 patients with RLS | Systematic Review and Meta-Analysis | The study reports a notable prevalence of RLS among patients who have experienced a stroke or TIA, indicating that RLS is a common post-stroke sleep disorder. RLS symptoms can emerge or worsen shortly after a stroke or TIA, possibly due to the acute impact on neural pathways and brain regions involved in sensory and motor control. | NA. |
| Ruppert et al. (2022) [59] | 16 patients with RLS | Prospective case study | The study highlights a significant prevalence of RLS in patients who have experienced a stroke, suggesting that stroke can be a trigger or exacerbating factor for RLS. Patients with stroke-related RLS may have slower recovery rates and poorer functional outcomes due to the additional burden of sleep disturbances. | NA. |
| Zorgor et al. (2022) [60] | 14 patients with RLS | Prospective cohort study | There is a significant occurrence of RLS in patients after experiencing an AIS. The location of the stroke lesion plays a crucial role in the development of RLS.<br><br>Patients with post-stroke RLS tend to exhibit more severe symptoms if the lesions are | 5.7% had post-stroke RLS. |

| | | | | | |
|---|---|---|---|---|---|
| | | located in the basal ganglia, which is involved in motor control. | | | |

Abbreviation: AIS: Acute Ischemic Stroke; CI: Confidence Interval; NIHSS: National Institute of Health Stroke Scale; OR: Odds Ratio; psRLS: Post-Stroke Restless Legs Syndrome.

# Table S8

**Table S8.** Management of sleep disorders associated with stroke.

| Treatment | Sleep disorder | Mechanism of action | Efficacy | Adverse effects | References |
|---|---|---|---|---|---|
| Modafinil/ armodafinil | OSA, EDS | Mainly as a catecholamine reuptake inhibitor | Reduced daytime sleepiness in men with mild to moderate OSA.<br>There is some benefit of modafinil for fatigue but no benefit for disability, cognition, and subscores of stroke-specific quality of life. | No significant adverse events. | Liu et al. (2024) [61]<br>Pacheco et al. (2019) [62] |
| Pitolisant | OSA, EDS | Selective H3 receptor antagonist/ inverse agonist | Evidence suggests a significant benefit of pitolisant in improving EDS and fatigue. | Insomnia, headache, abdominal discomfort, dizziness, anxiety, diarrhea, and nausea. | Liu et al. (2024) [61]<br>Leher et al. (2022) [63]<br>Arnulf et al. (2023) [64] |
| Solriamfetol | OSA, EDS | Selective dopamine and norepinephrine reuptake inhibitor | Solriamfetol is a first-line therapeutic agent to combat sleepiness in OSA and narcolepsy patients, and both the FDA and the EMA have approved it. | Headache decreased appetite;<br>Insomnia;<br>Nausea and anxiety. | Liu et al. (2024) [61]<br>Abad et al. (2021) [65] |
| Reboxetine (with oxybutynin) | OSA | Noradrenaline reuptake inhibitor | Reboxetine alone and combined with oxybutynin reduces OSA severity. | Among the most concerning side effects of reboxetine is the potential for aggressive behavior and suicidal ideation, though the potential for these behaviors exists | Liu et al. (2024) [61]<br>Altree et al. (2023) [66] |

| | | | | | |
|---|---|---|---|---|---|
| | | | | with many antidepressants. | |
| Atomoxetine (with oxybutynin) | OSA | Selective noradrenaline reuptake inhibitor | Atomoxetine, primarily used to treat OSA, has shown some effects related to stroke, though it is not commonly prescribed for stroke management. | It has been associated with significant cardiovascular risks, including stroke, especially in individuals with pre-existing heart conditions or high BP. | Liu et al. (2024) [61]<br>Corser et al. (2023) [67] |
| Oral iron | RLS | Unclear | Oral iron supplementation in stroke patients is primarily considered in the context of treating anemia, which is a common issue following a stroke. Anemia can exacerbate the recovery process and negatively impact overall outcomes. Iron deficiency, leading to anemia, can reduce oxygen delivery to tissues, including the brain, which is critical during the recovery phase post-stroke. | Oral iron is generally used due to its safety profile despite gastrointestinal side effects and less immediate effect compared to IV iron. | Gossard et al. (2021) [68]<br>Hanna-Rivero et al. (2022) [69]<br>Del Vecchio et al. (2020) [70] |
| Pramipexole, ropinirole, rotigotine, carbidopa/ levodopa | RLS | Dopamine agonists | Studies suggest that dopaminergic drugs can enhance motor recovery post-stroke, likely by promoting neural plasticity and functional recovery. However, the exact role and efficacy of carbidopa/levodopa in stroke rehabilitation require further investigation to confirm its benefits. Pramipexole may share some neuroprotective properties, but the exact role of stroke is limited by direct evidence. | Rotigotine<br>Common Side Effects:<br>Nausea<br>Application site reactions (for transdermal patch)<br>Dizziness<br>Drowsiness<br>Insomnia<br>Headache<br>Serious Side Effects:<br>Hallucinations<br>Compulsive behaviors<br>Orthostatic hypotension<br>Dyskinesia | Gossard et al. (2021) [68]<br>Andrabi et al. (2019) [71] |

| | | | | | |
|---|---|---|---|---|---|
| | | | | Carbidopa/Levodopa<br>Common Side Effects:<br>Nausea<br>Dizziness<br>Orthostatic hypotension<br>Dyskinesia<br>Insomnia<br>Dry mouth<br>Serious Side Effects:<br>Hallucinations<br>Depression<br>Sudden sleep episodes<br>Impulse control disorders<br>Confusion | |
| Gabapentin | RLS | Unclear | Gabapentin shows promise in aiding functional recovery after a stroke. It can enhance the recovery of motor functions by promoting neuronal plasticity, the brain's ability to reorganize itself and form new neural connections. | Common side effects include dizziness, drowsiness, and peripheral edema (swelling of the limbs). These symptoms can be particularly concerning for stroke patients as they may exacerbate balance issues and increase the risk of falls and further injury. | Gossard et al. (2021) [68]<br>Tedeschi et al. (2022) [72] |
| Tramadol | RLS | Opioid<br>It works by inhibiting the reuptake of serotonin and norepinephrine | Tramadol is used to manage moderate to moderately severe pain, which can be useful for post-stroke pain management, including central post-stroke pain (CPSP). It works by inhibiting the reuptake of serotonin and norepinephrine, which helps alleviate pain. | Tramadol is found to have no short-term increased risk of cardiovascular events, including ischemic stroke.<br>It may increase the risk of seizures, particularly in individuals with a history of epilepsy or prior stroke. | Gossard et al. (2021) [68]<br>Xie et al. (2021) [73] |
| Melatonin | Insomnia | MT1 and MT2 receptor agonists (in the suprachiasmatic nucleus) | Studies have shown that melatonin administration post-stroke improves motor coordination and reduces hyperactivity and anxiety, contributing | Common side effects of melatonin include dizziness, headaches, nausea, and daytime drowsiness. In stroke patients, there is an | Shinozuka et al. (2013) [74]<br>Sadanandan et al. (2020) [75] |

| | | | | | |
|---|---|---|---|---|---|
| | | | to better functional recovery. Melatonin has shown promising effects in the context of stroke, primarily due to its potent antioxidant and neuroprotective properties. | increased risk of confusion or disorientation, which could complicate their recovery process. | |
| Sodium oxybate | EDS | GABAB receptor agonist | Primarily used for treating narcolepsy, has implications for stroke risk due to its sodium content. It contributes significantly to daily sodium intake, ranging from 1100 to 1640 mg, which can elevate the risk of hypertension and cardiovascular diseases, including stroke. | Hypertension, hypernatremia, hyponatremia. | Bogan et al. (2021) [76]<br>Potasso et al. (2022) [77] |
| Lithium | EDS | Unclear | Some studies have indicated that lithium can improve motor recovery and cognitive function post-stroke. | It is important to note that lithium's therapeutic window is narrow, and its use requires careful monitoring to avoid toxicity. | Munteanu et al. (2022) [78] |
| Clonazepam | REM sleep behavior disorder | Benzodiazepine | Stroke patients may develop anxiety or have a higher risk of seizures, and clonazepam can be effective in managing these conditions due to its anxiolytic and anticonvulsant properties. | Common side effects include drowsiness, dizziness, and problems with coordination, which can exacerbate issues in stroke patients, such as fall risk and cognitive impairment. | Gilat et al. (2022) [79] |

Abbreviations: EDS: Excessive Daytime Sleepiness; EMA: European Medicines Agency; FDA: US Food and Drug Administration; OSA: Obstructive Sleep Apnea; RLS: Restless Legs Syndrome.

## References


1. Munoz, R.; Duran-Cantolla, J.; Martínez-Vila, E.; Gallego, J.; Rubio, R.; Aizpuru, F.; De La Torre, G. Severe Sleep Apnea and Risk of Ischemic Stroke in the Elderly. *Stroke* **2006**, *37*, 2317–2321, doi:10.1161/01.STR.0000236560.15735.0f.
2. Franklin, K.A.; Lindberg, E. Obstructive Sleep Apnea Is a Common Disorder in the Population-a Review on the Epidemiology of Sleep Apnea. *J Thorac Dis* **2015**, *7*, 1311–1322, doi:10.3978/j.issn.2072-1439.2015.06.11.
3. Morin, C.M.; Jarrin, D.C. Epidemiology of Insomnia: Prevalence, Course, Risk Factors, and Public Health Burden. *Sleep Med Clin* **2022**, *17*, 173–191, doi:10.1016/j.jsmc.2022.03.003.
4. Leng, Y.; Cappuccio, F.P.; Wainwright, N.W.J.; Surtees, P.G.; Luben, R.; Brayne, C.; Khaw, K.-T. Sleep Duration and Risk of Fatal and Nonfatal Stroke: A Prospective Study and Meta-Analysis. *Neurology* **2015**, *84*, 1072–1079, doi:10.1212/WNL.0000000000001371.
5. Liu, Y.; Liu, G.; Li, L.; Yang, J.; Ma, S. Evaluation of Cardiovascular Risk Factors and Restless Legs Syndrome in Women and Men: A Preliminary Population-Based Study in China. *J Clin Sleep Med* **2018**, *14*, 445–450, doi:10.5664/jcsm.6996.
6. Manconi, M.; Garcia-Borreguero, D.; Schormair, B.; Videnovic, A.; Berger, K.; Ferri, R.; Dauvilliers, Y. Restless Legs Syndrome. *Nat Rev Dis Primers* **2021**, *7*, 80, doi:10.1038/s41572-021-00311-z.
7. Ma, Y.; Wang, J.; Wang, Y.; Yang, G.-Y. The Biphasic Function of Microglia in Ischemic Stroke. *Prog Neurobiol* **2017**, *157*, 247–272, doi:10.1016/j.pneurobio.2016.01.005.
8. Dauvilliers, Y.; Schenck, C.H.; Postuma, R.B.; Iranzo, A.; Luppi, P.-H.; Plazzi, G.; Montplaisir, J.; Boeve, B. REM Sleep Behaviour Disorder. *Nat Rev Dis Primers* **2018**, *4*, 19, doi:10.1038/s41572-018-0016-5.
9. Hornyak, M.; Feige, B.; Riemann, D.; Voderholzer, U. Periodic Leg Movements in Sleep and Periodic Limb Movement Disorder: Prevalence, Clinical Significance and Treatment. *Sleep Med Rev* **2006**, *10*, 169–177, doi:10.1016/j.smrv.2005.12.003.
10. Plomaritis, P.; Theodorou, A.; Michalaki, V.; Stefanou, M.-I.; Palaiodimou, L.; Papagiannopoulou, G.; Kotsali-Peteinelli, V.; Bregianni, M.; Andreadou, E.; Paraskevas, G.P.; et al. Periodic Limb Movements during Sleep in Acute Stroke: Prevalence, Severity and Impact on Post-Stroke Recovery. *J Clin Med* **2023**, *12*, doi:10.3390/jcm12185881.
11. Coelho, F.M.S.; Georgsson, H.; Narayansingh, M.; Swartz, R.H.; Murray, B.J. Higher Prevalence of Periodic Limb Movements of Sleep in Patients with History of Stroke. *J Clin Sleep Med* **2010**, *6*, 428–430.
12. Mohsenin, V.; Valor, R. Sleep Apnea in Patients with Hemispheric Stroke. *Arch Phys Med Rehabil* **1995**, *76*, 71–76, doi:10.1016/s0003-9993(95)80046-8.

13. Arzt, M.; Young, T.; Finn, L.; Skatrud, J.B.; Bradley, T.D. Association of Sleep-Disordered Breathing and the Occurrence of Stroke. *Am J Respir Crit Care Med* **2005**, *172*, 1447–1451, doi:10.1164/rccm.200505-702OC.
14. Redline, S.; Yenokyan, G.; Gottlieb, D.J.; Shahar, E.; O@Connor, G.T.; Resnick, H.E.; Diener-West, M.; Sanders, M.H.; Wolf, P.A.; Geraghty, E.M.; et al. Obstructive Sleep Apnea-Hypopnea and Incident Stroke: The Sleep Heart Health Study. *Am J Respir Crit Care Med* **2010**, *182*, 269–277, doi:10.1164/rccm.200911-1746OC.
15. Young, T. Rationale, Design and Findings from the Wisconsin Sleep Cohort Study: Toward Understanding the Total Societal Burden of Sleep Disordered Breathing. *Sleep Med Clin* **2009**, *4*, 37–46, doi:10.1016/j.jsmc.2008.11.003.
16. Wang, X.; Ouyang, Y.; Wang, Z.; Zhao, G.; Liu, L.; Bi, Y. Obstructive Sleep Apnea and Risk of Cardiovascular Disease and All-Cause Mortality: A Meta-Analysis of Prospective Cohort Studies. *Int J Cardiol* **2013**, *169*, 207–214, doi:10.1016/j.ijcard.2013.08.088.
17. Hsieh, M.-S.; Hu, S.-Y.; How, C.-K.; Seak, C.-J.; Hsieh, V.C.-R.; Lin, J.-W.; Chen, P.-C. Hospital Outcomes and Cumulative Burden from Complications in Type 2 Diabetic Sepsis Patients: A Cohort Study Using Administrative and Hospital-Based Databases. *Ther Adv Endocrinol Metab* **2019**, *10*, 2042018819875406, doi:10.1177/2042018819875406.
18. Haula, T.-M.; Puustinen, J.; Takala, M.; Holm, A. Wake-up Strokes Are Linked to Obstructive Sleep Apnea and Worse Early Functional Outcome. *Brain Behav* **2021**, *11*, e2284, doi:10.1002/brb3.2284.
19. Edrissi, C.; Rathfoot, C.; Knisely, K.; Sanders, C.B.; Poupore, N.; Nathaniel, T. Gender Disparity in a Cohort of Stroke Patients with Incidence of Obstructive Sleep Apnea. *J Vasc Nurs* **2022**, *40*, 17–27, doi:10.1016/j.jvn.2021.10.002.
20. Robbins, R.; Yuan, Y.; Johnson, D.A.; Long, D.L.; Molano, J.; Kleindorfer, D.; Petrov, M.E.; Howard, V.J. Sleep Apnea and Incident Stroke in a National Cohort of Black and White Adults. *Neurology* **2024**, *102*, e209171, doi:10.1212/WNL.0000000000209171.
21. Elwood, P.; Hack, M.; Pickering, J.; Hughes, J.; Gallacher, J. Sleep Disturbance, Stroke, and Heart Disease Events: Evidence from the Caerphilly Cohort. *J Epidemiol Community Health* **2006**, *60*, 69–73, doi:10.1136/jech.2005.039057.
22. Jaussent, I.; Empana, J.-P.; Ancelin, M.-L.; Besset, A.; Helmer, C.; Tzourio, C.; Ritchie, K.; Bouyer, J.; Dauvilliers, Y. Insomnia, Daytime Sleepiness and Cardio-Cerebrovascular Diseases in the Elderly: A 6-Year Prospective Study. *PLoS One* **2013**, *8*, e56048, doi:10.1371/journal.pone.0056048.
23. Liu, Y.; Zhang, L.; Zhang, X.; Ma, J.; Jia, G. Effect of Combined Vagus Nerve Stimulation on Recovery of Upper Extremity Function in Patients with Stroke: A Systematic Review and Meta-Analysis. *J Stroke Cerebrovasc Dis* **2022**, *31*, 106390, doi:10.1016/j.jstrokecerebrovasdis.2022.106390.

24. Sands-Lincoln, M.; Loucks, E.B.; Lu, B.; Carskadon, M.A.; Sharkey, K.; Stefanick, M.L.; Ockene, J.; Shah, N.; Hairston, K.G.; Robinson, J.G.; et al. Sleep Duration, Insomnia, and Coronary Heart Disease among Postmenopausal Women in the Women⊗s Health Initiative. *J Womens Health (Larchmt)* **2013**, *22*, 477–486, doi:10.1089/jwh.2012.3918.
25. Wu, M.-P.; Lin, H.-J.; Weng, S.-F.; Ho, C.-H.; Wang, J.-J.; Hsu, Y.-W. Insomnia Subtypes and the Subsequent Risks of Stroke: Report from a Nationally Representative Cohort. *Stroke* **2014**, *45*, 1349–1354, doi:10.1161/STROKEAHA.113.003675.
26. Hsu, C.-Y.; Chen, Y.-T.; Chen, M.-H.; Huang, C.-C.; Chiang, C.-H.; Huang, P.-H.; Chen, J.-W.; Chen, T.-J.; Lin, S.-J.; Leu, H.-B.; et al. The Association Between Insomnia and Increased Future Cardiovascular Events: A Nationwide Population-Based Study. *Psychosom Med* **2015**, *77*, 743–751, doi:10.1097/PSY.0000000000000199.
27. Zheng, B.; Yu, C.; Lv, J.; Guo, Y.; Bian, Z.; Zhou, M.; Yang, L.; Chen, Y.; Li, X.; Zou, J.; et al. Insomnia Symptoms and Risk of Cardiovascular Diseases among 0.5 Million Adults: A 10-Year Cohort. *Neurology* **2019**, *93*, e2110–e2120, doi:10.1212/WNL.0000000000008581.
28. Xu, H.; Li, W.; Chen, J.; Zhang, P.; Rong, S.; Tian, J.; Zhang, Y.; Li, Y.; Cui, Z.; Zhang, Y. Associations between Insomnia and Large Vessel Occlusion Acute Ischemic Stroke: An Observational Study. *Clinics (Sao Paulo)* **2023**, *78*, 100297, doi:10.1016/j.clinsp.2023.100297.
29. Geusgens, C.A.V.; van Tilburg, D.C.H.; Fleischeuer, B.; Bruijel, J. The Relation between Insomnia and Depression in the Subacute Phase after Stroke. *Neuropsychol Rehabil* **2024**, 1–17, doi:10.1080/09602011.2024.2370072.
30. Janes, F.; Lorenzut, S.; Bevilacqua, F.; de Biase, S.; Zilli, M.; Gigli, G.L.; Valente, M. Cerebrovascular Risk in Restless Legs Syndrome: Intima-Media Thickness and Cerebral Vasomotor Reactivity: A Case-Control Study. *Nat Sci Sleep* **2021**, *13*, 967–975, doi:10.2147/NSS.S302749.
31. Kalampokini, S.; Hommel, A.L.A.J.; Lorenzl, S.; Ferreira, J.J.; Meissner, W.G.; Odin, P.; Bloem, B.R.; Dodel, R.; Schrag, A.-E. Caregiver Burden in Late-Stage Parkinsonism and Its Associations. *J Geriatr Psychiatry Neurol* **2022**, *35*, 110–120, doi:10.1177/0891988720968263.
32. Schlesinger, I.; Erikh, I.; Nassar, M.; Sprecher, E. Restless Legs Syndrome in Stroke Patients. *Sleep Med* **2015**, *16*, 1006–1010, doi:10.1016/j.sleep.2014.12.027.
33. Young, T.; Palta, M.; Dempsey, J.; Skatrud, J.; Weber, S.; Badr, S. The Occurrence of Sleep-Disordered Breathing among Middle-Aged Adults. *N Engl J Med* **1993**, *328*, 1230–1235, doi:10.1056/NEJM199304293281704.
34. Seiler, A.; Camilo, M.; Korostovtseva, L.; Haynes, A.G.; Brill, A.-K.; Horvath, T.; Egger, M.; Bassetti, C.L. Prevalence of Sleep-Disordered Breathing after Stroke and

TIA: A Meta-Analysis. *Neurology* **2019**, *92*, e648–e654, doi:10.1212/WNL.0000000000006904.

35. Hermann, D.M.; Bassetti, C.L. Role of Sleep-Disordered Breathing and Sleep-Wake Disturbances for Stroke and Stroke Recovery. *Neurology* **2016**, *87*, 1407–1416, doi:10.1212/WNL.0000000000003037.
36. Gan-Or, Z.; Alcalay, R.N.; Rouleau, G.A.; Postuma, R.B. Sleep Disorders and Parkinson Disease; Lessons from Genetics. *Sleep Med Rev* **2018**, *41*, 101–112, doi:10.1016/j.smrv.2018.01.006.
37. Kamat, D.; Al-Ajlouni, Y.A.; Hall, R.C.W. The Therapeutic Impact of Plant-Based and Nutritional Supplements on Anxiety, Depressive Symptoms and Sleep Quality among Adults and Elderly: A Systematic Review of the Literature. *Int J Environ Res Public Health* **2023**, *20*, doi:10.3390/ijerph20065171.
38. Ancoli-Israel, S.; Walsh, J.K.; Mangano, R.M.; Fujimori, M. Zaleplon, A Novel Nonbenzodiazepine Hypnotic, Effectively Treats Insomnia in Elderly Patients Without Causing Rebound Effects. *Prim Care Companion J Clin Psychiatry* **1999**, *1*, 114–120, doi:10.4088/pcc.v01n0404.
39. National Institutes of Health State of the Science Conference Statement on Manifestations and Management of Chronic Insomnia in Adults, June 13-15, 2005. *Sleep* **2005**, *28*, 1049–1057, doi:10.1093/sleep/28.9.1049.
40. Hepburn, M.; Bollu, P.C.; French, B.; Sahota, P. Sleep Medicine: Stroke and Sleep. *Mo Med* **2018**, *115*, 527–532.
41. Vock, J.; Achermann, P.; Bischof, M.; Milanova, M.; Müller, C.; Nirkko, A.; Roth, C.; Bassetti, C.L. Evolution of Sleep and Sleep EEG after Hemispheric Stroke. *J Sleep Res* **2002**, *11*, 331–338, doi:10.1046/j.1365-2869.2002.00316.x.
42. Jang, S.H.; Chang, C.H.; Jung, Y.J.; Seo, J.P. Post-Stroke Hypersomnia. *Int J Stroke* **2016**, *11*, NP5-6, doi:10.1177/1747493015607502.
43. Hermann, D.M.; Bassetti, C.L. Sleep-Related Breathing and Sleep-Wake Disturbances in Ischemic Stroke. *Neurology* **2009**, *73*, 1313–1322, doi:10.1212/WNL.0b013e3181bd137c.
44. Sechi, G.; Agnetti, V.; Galistu, P.; Murgia, B.; Marrosu, F.; Puligheddu, M.; Conti, M.; Paulus, K.S. Restless Legs Syndrome and Periodic Limb Movements after Ischemic Stroke in the Right Lenticulostriate Region. *Parkinsonism Relat Disord* **2008**, *14*, 157–160, doi:10.1016/j.parkreldis.2007.02.004.
45. Paolucci, S. Epidemiology and Treatment of Post-Stroke Depression. *Neuropsychiatr Dis Treat* **2008**, *4*, 145–154, doi:10.2147/ndt.s2017.
46. Boulos, M.I.; Wan, A.; Im, J.; Elias, S.; Frankul, F.; Atalla, M.; Black, S.E.; Basile, V.S.; Sundaram, A.; Hopyan, J.J.; et al. Identifying Obstructive Sleep Apnea after Stroke/TIA: Evaluating Four Simple Screening Tools. *Sleep Med* **2016**, *21*, 133–139, doi:10.1016/j.sleep.2015.12.013.

47. Dharmakulaseelan, L.; Black, S.E.; Swartz, R.H.; Murray, B.J.; Boulos, M.I. Sex Differences in Obstructive Sleep Apnea after Stroke. *Can J Neurol Sci* **2024**, *51*, 557–563, doi:10.1017/cjn.2023.300.
48. Klingman, K.J.; Billinger, S.A.; Britton-Carpenter, A.; Bartsch, B.; Duncan, P.W.; Fulk, G.D. Prevalence and Detection of Obstructive Sleep Apnea Early after Stroke. *medRxiv : the preprint server for health sciences* 2024, 2024.06.16.24309011.
49. Rangel, M.F. de A.; Silva, L.C.; Gonçalves, E.H.; Silva, A.; Teixeira-Salmela, L.F.; Scianni, A.A. Presence of Self-Reported Sleep Alterations After Stroke and Their Relationship With Disability: A Longitudinal Study. *Neurorehabil Neural Repair* **2024**, *38*, 518–526, doi:10.1177/15459683241252826.
50. Yang, P.; Shi, M.; Jia, Y.; Zhong, C.; Peng, H.; Sun, L.; Guo, D.; Chen, J.; Wang, A.; Xu, T.; et al. Plasma Polyamines and Short-Term Adverse Outcomes Among Patients With Ischemic Stroke: A Prospective Cohort Study. *J Am Heart Assoc* **2024**, *13*, e035837, doi:10.1161/JAHA.124.035837.
51. Yang, H.-L.; Wang, M.; Xu, Y.-F.; Mo, B.-R.; Liu, X.-L.; Redding, S.R. Understanding Adherence to Continuous Positive Airway Pressure in Patients with Obstructive Sleep Apnea Post-Stroke: A Prospective Study Based on the Andersen Model. *Nurs Health Sci* **2024**, *26*, e13129, doi:10.1111/nhs.13129.
52. Boulos, M.I.; Wan, A.; Black, S.E.; Lim, A.S.; Swartz, R.H.; Murray, B.J. Restless Legs Syndrome after High-Risk TIA and Minor Stroke: Association with Reduced Quality of Life. *Sleep Med* **2017**, *37*, 135–140, doi:10.1016/j.sleep.2017.05.020.
53. Gupta, A.; Shukla, G.; Mohammed, A.; Goyal, V.; Behari, M. Restless Legs Syndrome, a Predictor of Subcortical Stroke: A Prospective Study in 346 Stroke Patients. *Sleep Med* **2017**, *29*, 61–67, doi:10.1016/j.sleep.2015.05.025.
54. Woo, H.G.; Lee, D.; Hwang, K.J.; Ahn, T.-B. Post-Stroke Restless Leg Syndrome and Periodic Limb Movements in Sleep. *Acta Neurol Scand* **2017**, *135*, 204–210, doi:10.1111/ane.12582.
55. Shiina, T.; Suzuki, K.; Okamura, M.; Matsubara, T.; Hirata, K. Restless Legs Syndrome and Its Variants in Acute Ischemic Stroke. *Acta Neurol Scand* **2019**, *139*, 260–268, doi:10.1111/ane.13055.
56. Han, S.-H.; Park, K.-Y.; Kim, J.-M.; Youn, Y.C.; Shin, H.-W. Restless Legs Syndrome Is Associated with Arterial Stiffness and Clinical Outcome in Stroke Patients. *Sleep Med* **2019**, *60*, 219–223, doi:10.1016/j.sleep.2019.03.027.
57. Zhang, L.; Meng, R.; Shang, S.; Wu, C.; Wu, D.; Shang, S.; Chen, L.; Zhang, Y.; Ji, X. Obstructive Sleep Apnea before Ischemic Stroke: Clinical Relevance to Infarction Volume and Neurological Recovery. *J Stroke Cerebrovasc Dis* **2019**, *28*, 2132–2139, doi:10.1016/j.jstrokecerebrovasdis.2019.04.008.
58. Hasan, F.; Gordon, C.; Wu, D.; Huang, H.-C.; Yuliana, L.T.; Susatia, B.; Marta, O.F.D.; Chiu, H.-Y. Dynamic Prevalence of Sleep Disorders Following Stroke or

Transient Ischemic Attack: Systematic Review and Meta-Analysis. *Stroke* **2021**, *52*, 655–663, doi:10.1161/STROKEAHA.120.029847.
59. Ruppert, E.; Hacquard, A.; Tatu, L.; Namer, I.J.; Wolff, V.; Kremer, S.; Lagha-Boukbiza, O.; Bataillard, M.; Bourgin, P. Stroke-Related Restless Legs Syndrome: Clinical and Anatomo-Functional Characterization of an Emerging Entity. *Eur J Neurol* **2022**, *29*, 1011–1016, doi:10.1111/ene.15207.
60. Zorgor, G.; Kabeloglu, V.; Soysal, A. Restless Legs Syndrome after Acute Ischemic Stroke and Its Relation to Lesion Location. *Sleep Biol Rhythms* **2022**, *20*, 551–560, doi:10.1007/s41105-022-00401-9.
61. Liu, J.; Yang, X.; Li, G.; Liu, P. Pharmacological Interventions for the Treatment of Obstructive Sleep Apnea Syndrome. *Front Med (Lausanne)* **2024**, *11*, 1359461, doi:10.3389/fmed.2024.1359461.
62. Pacheco, R.L.; Latorraca, C. de O.C.; da Silva, L.D.G.M.; Ferreira, D.B.G. de M.; Fernandes, C. de A.A.; Hosni, N.D.; Cabrera Martimbianco, A.L.; Vianna Pachito, D.; Riera, R. Modafinil for Poststroke Patients: A Systematic Review. *Int J Clin Pract* **2019**, *73*, e13295, doi:10.1111/ijcp.13295.
63. Leher, I.; Fleischmann, C.; Scherb, D.; Kollerer, M.; Miehling, J.; Wartzack, S.; Sesselmann, S. Patient-Specific Modelling for Preoperative Estimation of Hip Mechanics for Improved Planning of Total Hip Endoprosthesis Using Multibody Simulations.; Springer, 2021; pp. 1088–1096.
64. Arnulf, I.; Thomas, R.; Roy, A.; Dauvilliers, Y. Update on the Treatment of Idiopathic Hypersomnia: Progress, Challenges, and Expert Opinion. *Sleep Med Rev* **2023**, *69*, 101766, doi:10.1016/j.smrv.2023.101766.
65. Abad, V.C. Profile of Solriamfetol in the Management of Excessive Daytime Sleepiness Associated with Narcolepsy or Obstructive Sleep Apnea: Focus on Patient Selection and Perspectives. *Nat Sci Sleep* **2021**, *13*, 75–91, doi:10.2147/NSS.S245020.
66. Altree, T.J.; Aishah, A.; Loffler, K.A.; Grunstein, R.R.; Eckert, D.J. The Norepinephrine Reuptake Inhibitor Reboxetine Alone Reduces Obstructive Sleep Apnea Severity: A Double-Blind, Placebo-Controlled, Randomized Crossover Trial. *J Clin Sleep Med* **2023**, *19*, 85–96, doi:10.5664/jcsm.10256.
67. Corser, B.; Eves, E.; Warren-McCormick, J.; Rucosky, G. Effects of Atomoxetine plus a Hypnotic on Obstructive Sleep Apnea Severity in Patients with a Moderately Collapsible Pharyngeal Airway. *J Clin Sleep Med* **2023**, *19*, 1035–1042, doi:10.5664/jcsm.10464.
68. Gossard, T.R.; Trotti, L.M.; Videnovic, A.; St Louis, E.K. Restless Legs Syndrome: Contemporary Diagnosis and Treatment. *Neurotherapeutics* **2021**, *18*, 140–155, doi:10.1007/s13311-021-01019-4.

69. Hanna-Rivero, N.; Tu, S.J.; Elliott, A.D.; Pitman, B.M.; Gallagher, C.; Lau, D.H.; Sanders, P.; Wong, C.X. Anemia and Iron Deficiency in Patients with Atrial Fibrillation. *BMC Cardiovasc Disord* **2022**, *22*, 204, doi:10.1186/s12872-022-02633-6.
70. Del Vecchio, L.; Minutolo, R. ESA, Iron Therapy and New Drugs: Are There New Perspectives in the Treatment of Anaemia? *J Clin Med* **2021**, *10*, doi:10.3390/jcm10040839.
71. Andrabi, S.S.; Ali, M.; Tabassum, H.; Parveen, S.; Parvez, S. Pramipexole Prevents Ischemic Cell Death via Mitochondrial Pathways in Ischemic Stroke. *Dis Model Mech* **2019**, *12*, doi:10.1242/dmm.033860.
72. Tedeschi, A.; Larson, M.J.E.; Zouridakis, A.; Mo, L.; Bordbar, A.; Myers, J.M.; Qin, H.Y.; Rodocker, H.I.; Fan, F.; Lannutti, J.J.; et al. Harnessing Cortical Plasticity via Gabapentinoid Administration Promotes Recovery after Stroke. *Brain* **2022**, *145*, 2378–2393, doi:10.1093/brain/awac103.
73. Xie, J.; Strauss, V.Y.; Martinez-Laguna, D.; Carbonell-Abella, C.; Diez-Perez, A.; Nogues, X.; Collins, G.S.; Khalid, S.; Delmestri, A.; Turkiewicz, A.; et al. Association of Tramadol vs Codeine Prescription Dispensation With Mortality and Other Adverse Clinical Outcomes. *JAMA* **2021**, *326*, 1504–1515, doi:10.1001/jama.2021.15255.
74. Shinozuka, K.; Staples, M.; Borlongan, C.V. Melatonin-Based Therapeutics for Neuroprotection in Stroke. *Int J Mol Sci* **2013**, *14*, 8924–8947, doi:10.3390/ijms14058924.
75. Sadanandan, N.; Cozene, B.; Cho, J.; Park, Y.J.; Saft, M.; Gonzales-Portillo, B.; Borlongan, C.V. Melatonin-A Potent Therapeutic for Stroke and Stroke-Related Dementia. *Antioxidants (Basel)* **2020**, *9*, doi:10.3390/antiox9080672.
76. Bogan, R.K.; Thorpy, M.J.; Dauvilliers, Y.; Partinen, M.; Del Rio Villegas, R.; Foldvary-Schaefer, N.; Skowronski, R.; Tang, L.; Skobieranda, F.; Šonka, K. Efficacy and Safety of Calcium, Magnesium, Potassium, and Sodium Oxybates (Lower-Sodium Oxybate [LXB]; JZP-258) in a Placebo-Controlled, Double-Blind, Randomized Withdrawal Study in Adults with Narcolepsy with Cataplexy. *Sleep* **2021**, *44*, doi:10.1093/sleep/zsaa206.
77. Potasso, L.; Refardt, J.; De Marchis, G.M.; Wiencierz, A.; Wright, P.R.; Wagner, B.; Dittrich, T.; Polymeris, A.A.; Gensicke, H.; Bonati, L.H.; et al. Impact of Sodium Levels on Functional Outcomes in Patients With Stroke - A Swiss Stroke Registry Analysis. *J Clin Endocrinol Metab* **2022**, *107*, e672–e680, doi:10.1210/clinem/dgab650.
78. Munteanu, C.; Rotariu, M.; Turnea, M.; Tătăranu, L.G.; Dogaru, G.; Popescu, C.; Spînu, A.; Andone, I.; Ionescu, E.V.; Țucmeanu, R.E.; et al. Lithium Biological Action Mechanisms after Ischemic Stroke. *Life (Basel)* **2022**, *12*, doi:10.3390/life12111680.
79. Gilat, M.; Marshall, N.S.; Testelmans, D.; Buyse, B.; Lewis, S.J.G. A Critical Review of the Pharmacological Treatment of REM Sleep Behavior Disorder in Adults: Time

for More and Larger Randomized Placebo-Controlled Trials. *J Neurol* **2022**, *269*, 125–148, doi:10.1007/s00415-020-10353-0.
